# The family Raphitomidae (Mollusca: Gastropoda: Conoidea) in the Greek Seas with the description of two new species

**DOI:** 10.1186/s40709-018-0085-3

**Published:** 2018-07-09

**Authors:** Thanasis Manousis, Constantinos Kontadakis, George Mbazios, Georgios Polyzoulis

**Affiliations:** 1 Epanomi, Greece; 2 Athens, Greece; 3 Athens, Greece; 4 Redestos, Greece

**Keywords:** Raphitomidae, Mediterranean Sea, New *Raphitoma* species, Greece

## Abstract

**Background:**

The Raphitomidae family in the Mediterranean Sea is under revision. Accordingly, new data are of taxonomic and comparative relevance. In this study, new material from the Hellenic Seas is presented.

**Results:**

The Raphitomidae fauna of Greece was collected and investigated during the period from October 2008 to February 2018. Thirty-five (35) species were identified and their status was compared with existing checklists and other collections. This effort revealed two new *Raphitoma* species, and one new record for the Mediterranean Sea. Also from the present collection, four species are new records for the East Mediterranean, 10 for the Hellenic fauna and six are reported for second time. The main identification characteristics and baseline ecological information are given and discussed.

**Conclusions:**

By this report, the Hellenic Raphitomidae biodiversity is enriched by 10 new records, out of which, two are new species, one is new record for the Mediterranean Sea, and four for the East basin.

## Background

The Mediterranean Sea is rich in biodiversity; the estimated number of species raised from 8500 (two decades ago) [1] to more than 17,000 species recently [2]. More than 2100 of them are molluscs, among which 1673 are gastropods [3].

Conoidea is a diverse superfamily of venomous and exclusively marine gastropods within the Neogastropoda, which harbours more than 300 genera, 4000 known species and an estimated number of over 12,000 existing species [4–6]. Due to the species richness and the extensive homoplasy among shell’s features and the anterior alimentary system, their classification remained problematic; relevant attempts have been hindered primarily by the absence of a stable phylogenetic framework. Recently, the taxonomy of Conoidea has been put on a firmer background based mainly on extensive new material from the tropical Western Pacific. This has led to both a detailed molecular phylogeny of the entire superfamily [7–9] and to several generic revisions (e.g. [10–14]). One of the outcomes of those works has been that the traditional “family” Turridae (sensu Powell 1966) [15] was split into 13 monophyletic families of which the Raphitomidae is the largest and most diverse.

The Raphitomidae of the Mediterranean Sea appears as a morphologically homogeneous group of rather common infralittoral inhabitants readily recognizable by the sculpture and color pattern of the shell. The ongoing revision of the genus *Raphitoma* in the Mediterranean Sea by Pusateri et al. [16–20] led to 40 species. This latter revision, together with the check list by Cachia et al. [21] that contains also brief descriptions, and the presentation of certain species by Gofas et al. [22] seem to be the best taxonomic guides of the group, with eight new species described so far.

A comprehensive survey of the distribution of *Raphitoma* species faces a number of difficulties [16, 17], mainly due to the large variability in the shell morphological characters and the extensive overlap in morphology between closely related species. The recent revision of the Mediterranean *Raphitoma* species has been mainly based on differences in the size and shape of their protoconch [16, 17], following the assumption that the dichotomy “multispiral protoconch/planktotrophic development vs. paucispiral protoconch/lecithotrophic development” [23] could be used in Caenogastropoda to recognise the sister species [24–27]. Peocilogony has been recently proposed to explain the existence of Mediterranean “sibling” *Raphitoma* species [28].

Considering all the above, the aim of this study was to contribute to the classification of the Raphitomidae family by presenting new material from the Hellenic Seas.

## Results

### The records

Overall 568 specimens were collected identified to 35 species. Out of them, two are recognized as new *Raphitoma* species. Another one species is a new record for the Mediterranean Sea, and other four species are new for the East Mediterranean basin. Overall, 10 species are new records for the Hellenic fauna, and other six are second records.

#### The records of the Hellenic fauna

All records of Raphitomidae from the Hellenic waters are presented below within genuses. For the new and second records, the collection stations, the species’ description, the habitat and geographic distribution, the status of the species together with its 1st and/or 2nd documented records are given. Species images are also provided in support to the identification of Greek raphitomids.

Systematics

Superfamily **Conoidea** Fleming, 1822

Family **Raphitomidae** Bellardi, 1875

Genus: ***Clathromangelia*** Monterosato, 1884

Type species *Pleurotoma granum* Philippi, 1844 by monotypy

**Fig. 1:**
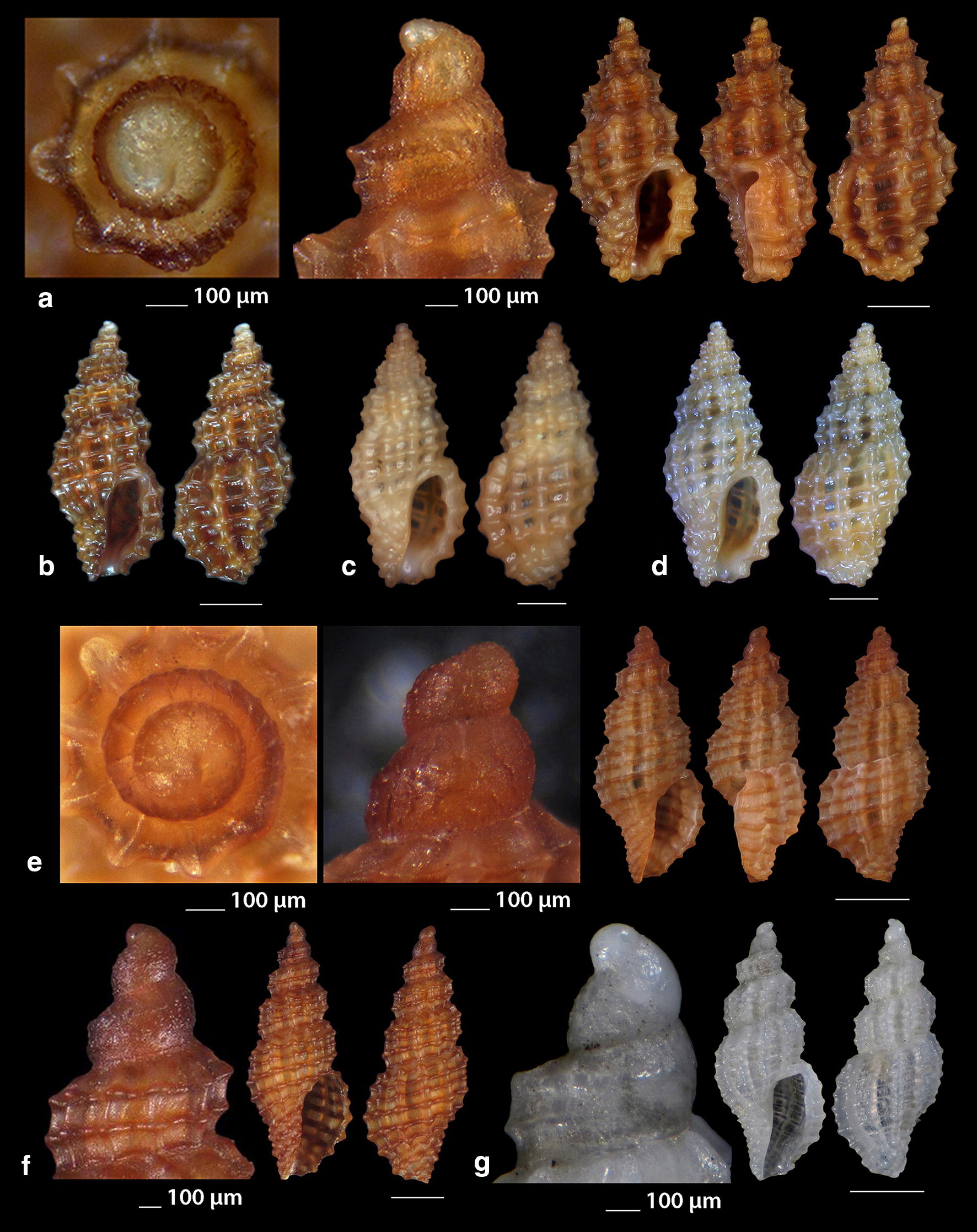
**a**–**d** *Clathromangelia granum*, **e**–**g** *Clathromangelia loiselieri*. Bar = 1 mm, unless otherwise indicated

***Clathromangelia granum*** (Philippi, 1844) (Fig. 1a–d)

*Collection stations* 102 shells (4.20–5.25 mm long, 2.00–2.45 mm wide), − 40 m, Anafi Island, 36°20′N–25°46′E; − 6/8 m, mixed bottoms with *Posidonia oceanica*, Amoopi, Karpathos Island, 35°28′N–27°11′Ε; Diakoftis, Karpathos Island, 35°24′N–27°09′Ε; − 3 m, mixed bottom with *Posidonia oceanica*, Pantokratoras, Preveza, 38°94′N–20°73′E.

*Description* Shell hyaline, biconic, robust and nearly 2.2 times as long as wide. Pointed multispiral protoconch, of mean 475 μm wide and 580 μm high, 1.75 highly cancellated and convex whorls of a variety of sculptures in accordance with the description of Oliverio [26]. The cancellation starts straight from the nucleus while the last whorl exhibits a rather strong keel before the onset of the teleoconch. Protoconch I of approximately 0.7 whorls and mean 305 μm wide. Teleoconch of four convex whorls characterised by 9–10 solid and ortho- to slightly prosocline axial ribs on the body whorl and nine conspicuous and equally spaced spiral ridges that endow the shell with a gross reticulated appearance. The body whorl occupies almost 65% of the total length while the aperture slightly more than 45%, and is quadrangular with a short and wide anterior and a deep posterior siphonal canals. The columella is smooth and sinuous. Outer lip particularly thickened with six prominent outer and three weak internal teeth. Background color uniformly light beige to honey-brown with more light axial ribs.

*Similar species* It differs from *C. loiselieri* Oberling, 1970 in that is bigger, wider and with less axial ribs. It also differs from *C. strigilata* Pallary, 1904 in its less small spiral ridges [29].

*Habitat and distribution* Detrital and muddy bottoms and in caves of the entire Mediterranean Sea [29–31].

*Status* Common [31]. Second documented record for the Hellenic Seas [32].

***Clathromangelia loiselieri*** Oberling, 1970 (Fig. 1e–g)

*Collection stations* 96 shells (3.65–5.00 mm long, 1.55–2.00 mm wide), − 10 m, mixed bottom, Paralia, Epanomi, 40°26′N–22°48′Ε; − 50/60 m, Skyros Island, 38°50′N–24°30′Ε; − 80/120 m, hard substrate, Lavrio, Attiki, 37°41′N–24°06′E and Central Saronikos Gulf, 37°44′N–23°48′Ε;—60/120 m, Kythnos Island, 37°27′N–24°27′Ε; − 50/70 m, Astypalaia Island 36°31′N–26°26′E; Amoopi, Karpathos Island, 35°28′N–27°11′Ε; Dakoftis, Karpathos Island, 35°24′N–27°09′Ε; Lefkos, Karpathos Island, 35°37′N–27°04′Ε; − 6/8 m, Balos, Crete, 35°35′N–23°35′E; − 120/160 m, Gytheio, Laconia, 36°44′N–22°36′E; − 60/80 m, Kardamili, Messinia, 36°51′N–22°13′E.

*Description* Shell hyaline, biconic, delicate and nearly 2.5 times as long as wide. Pointed and slender multispiral protoconch, mean 440 μm wide, 660 μm high, 2.25 evenly granulate and convex whorls with two weak keels before the onset of the teleoconch. Protoconch I of approximately 1 whorl and mean 260 μm wide. Teleoconch of four moderately convex whorls with 11–12 narrow axial ribs as wide as the interspaces on the body whorl and 10 also narrow and rather equally spaced spiral cords that endow the shell with a delicate reticulated appearance. The body whorl occupies almost 65% of the total length while the aperture slightly more than 40% and is oval with a short and wide anterior and a deep posterior siphonal canals. The columella is smooth and sinuous. Outer lip lightly thickened with 6 outer and 5 weak internal teeth. Background color uniformly light honey-brown with darker the spiral cords and the apex. Pure white also, but less frequent.

*Similar species C. loiselieri* differs from *C. granum* (Philippi, 1844) in that is slender and with more axial ribs. It also differs from *C. strigilata* Pallary, 1904 in that it is larger and much slender [29, 33].

*Habitat and distribution* Detrital and muddy bottoms of the East Mediterranean Sea [29, 31]. This is a second record as the species was referred without description from the Hellenic waters—Saronikos Gulf [26].

*Status* Rare [31]. Second documented record for the Hellenic Seas [32].

**Fig. 2:**
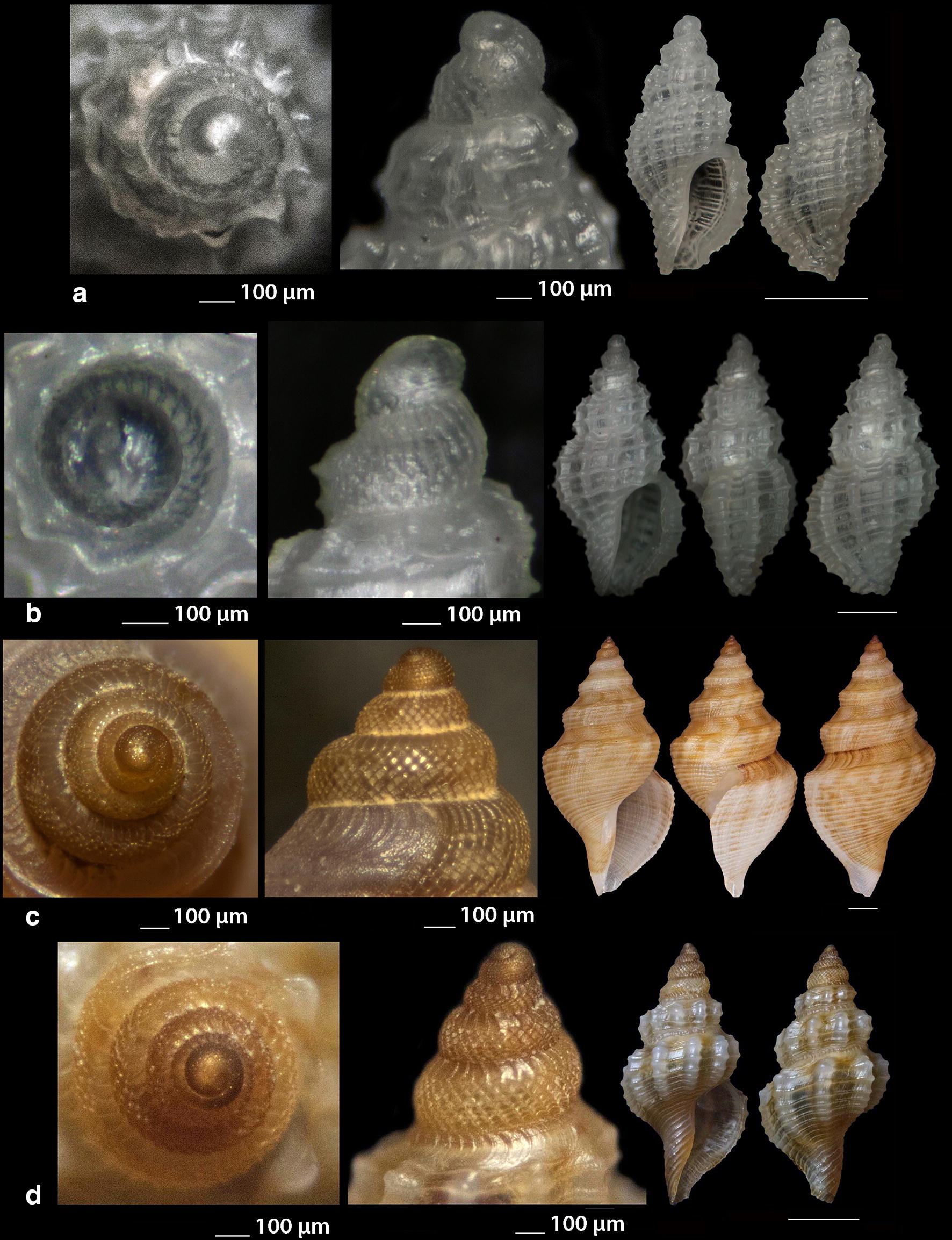
**a**, **b** *Clathromangelia strigilata*, **c** *Gymnobela abyssorum*, **d** *Pleurotomella gibbera*. Bar = 1 mm, unless otherwise indicated

***Clathromangelia strigilata*** Pallary, 1904 (Fig. 2a, b)

*Collection stations* Six shells (3.05–4.35 mm long, 1.40–2.20 mm wide), − 60/70 m, biogenic bottoms, Lemnos Island, 40°05′N–25°12′E; − 6/8 m, Balos, Crete, 35°35′N–23°35′E.

*Description* Shell hyaline, biconic, inflated and nearly 2.2 times as long as wide. Papilla shaped multispiral protoconch, 430 μm (mean) wide and mean 480 μm high with 1.75 cancellated and convex whorls and 1 weak keel before the onset of the teleoconch. Protoconch I of approximately 0.75 whorls and mean 290 μm in diameter. Teleoconch of 3 highly convex whorls with 10 narrow axial ribs half as wide as the interspaces on the body whorl and 14 also narrow and unequally spaced spiral cords that endow the shell with a delicate reticulated appearance. The body whorl occupies some more than 60% of the total length while the aperture some more than 45% and is oval with a short and wide anterior and a deep posterior siphonal canals. The columella is smooth and sinuous. Outer lip lightly thickened with 10 outer teeth. Uniformly milk-white in color.

*Similar species C. strigilata* differs from *C. granum* in that is smaller, inflated and with more axial ribs and spiral ridges. It also differs from *C. loiselieri* in that it is much smaller and much more inflated [33, 34].

*Habitat and distribution* Detrital and muddy bottoms of the South and Central Mediterranean Sea [31, 34].

*Status* Rare [31]. Second documented record for the Hellenic Seas [32].

Genus: ***Gymnobela*** Verrill, 1884

Type species *Gymnobela engonia* Verrill, 1884 by subsequent designation Cossmann, 1896

***Gymnobela abyssorum*** (Locard, 1897) (Fig. 2c)

*Collection station* One shell (9.00 mm long, 4.50 mm wide) biogenic material, − 400 m, Lemnos Island, 40°05′N–25°12′E.

*Description* Shell biconic, inflated, scalariform and nearly 2 times as long as wide. Protoconch comparatively large, multispiral, nearly 650 μm wide, 900 μm high, of 4.50 highly cancellated and convex whorls. The cancellation starts straight from the nucleus. Teleoconch with 4 angular whorls exhibiting a blunt keel bordered in its adapical part by a shallow groove and a strong shoulder immediately below a shallow suture. The surface below the suture is ornamented with fine spiral striae and forms a slope towards the keel. The body whorl occupies some more than 65% of the length and bears 13–14 oblique, short and inconspicuous axial ribs limited to the middle part of the whorls, with interspaces approximately as wide as the ribs. Spiral decoration formed by spiral cordlets that are slightly marked and flattened. The aperture occupies approximately 50% of the shell length, is quadrangular and with a short tail. Columella smooth and sinuous. Outer lip simple, sharp and curved at side view, with a wide sinus adapically. Background color beige with sinuοus and diffuse light brown radial smudges arranged in a regular manner. Protoconch light brown.

*Similar species* Easily distinguishable even when immature due to its characteristic large protoconch and sculpture [22, 35].

*Habitat and distribution* Circalittoral and bathyal muddy bottoms of the Mediterranean Sea, below 200 m [22, 31, 35].

*Status* Rare [31]. Second documented record for the Hellenic Seas [32].

Genus: ***Pleurotomella*** Verrill, 1872

Type species *Pleurotomella packardi* Verrill, 1872 by monotypy

***Pleurotomella gibbera*** Bouchet & Warén, 1980 (Fig. 2d)

*Collection station* Two shells (2.65 and 3.80 mm long, 1.25 and 1.85 mm wide, respectively) coralligenous hard substrate, − 400 m, Lemnos Island, 40°05′N–25°12′E.

*Description* Large hyaline and translucent multispiral protoconch, 650 μm wide, 670 μm high, of 4.5 highly cancellated and convex whorls. Protoconch I of 1.1 whorls and 175 μm in diameter. The cancellation starts straight from the nucleus while the last whorl exhibits 2 weak keels before the onset of the protoconch. Teleoconch with 1 conspicuously convex whorl exhibiting an oblique shoulder immediately below a shallow suture and 10 strong orthocline axial ribs with interspaces slightly wider than the ribs. Spiral decoration of 20 spiral cords that become denser towards the shell’s tail and forming inconspicuous tubercles at the crossings with the ribs. Aperture elongated and with a long anterior canal. The columella is smooth and sinuous. Shell cream-white, with a beige protoconch that is more intense at the apical whorls.

*Similar species* Easily distinguishable from its congenerates by its characteristic protoconch [22].

*Habitat and distribution* Coralligenous bottoms from 100 to 400 m of the Mediterranean Sea [22, 31]. *Pleurotomella gibbera* is referred as a species of a rather restricted distribution from the Central and East Atlantic to the West and Central Mediterranean Sea [36].

*Status* Rare [31]. First documented record for the Hellenic Seas.

Genus: ***Raphitoma*** Bellardi, 1847

*Type species Pleurotoma hystrix* Cristofori & Jan, 1832 [nomen nudum, validated by Bellardi [37] as “*Pleurotoma histrix Jan.*”], subsequently designated by Monterosato [38].

**Fig. 3:**
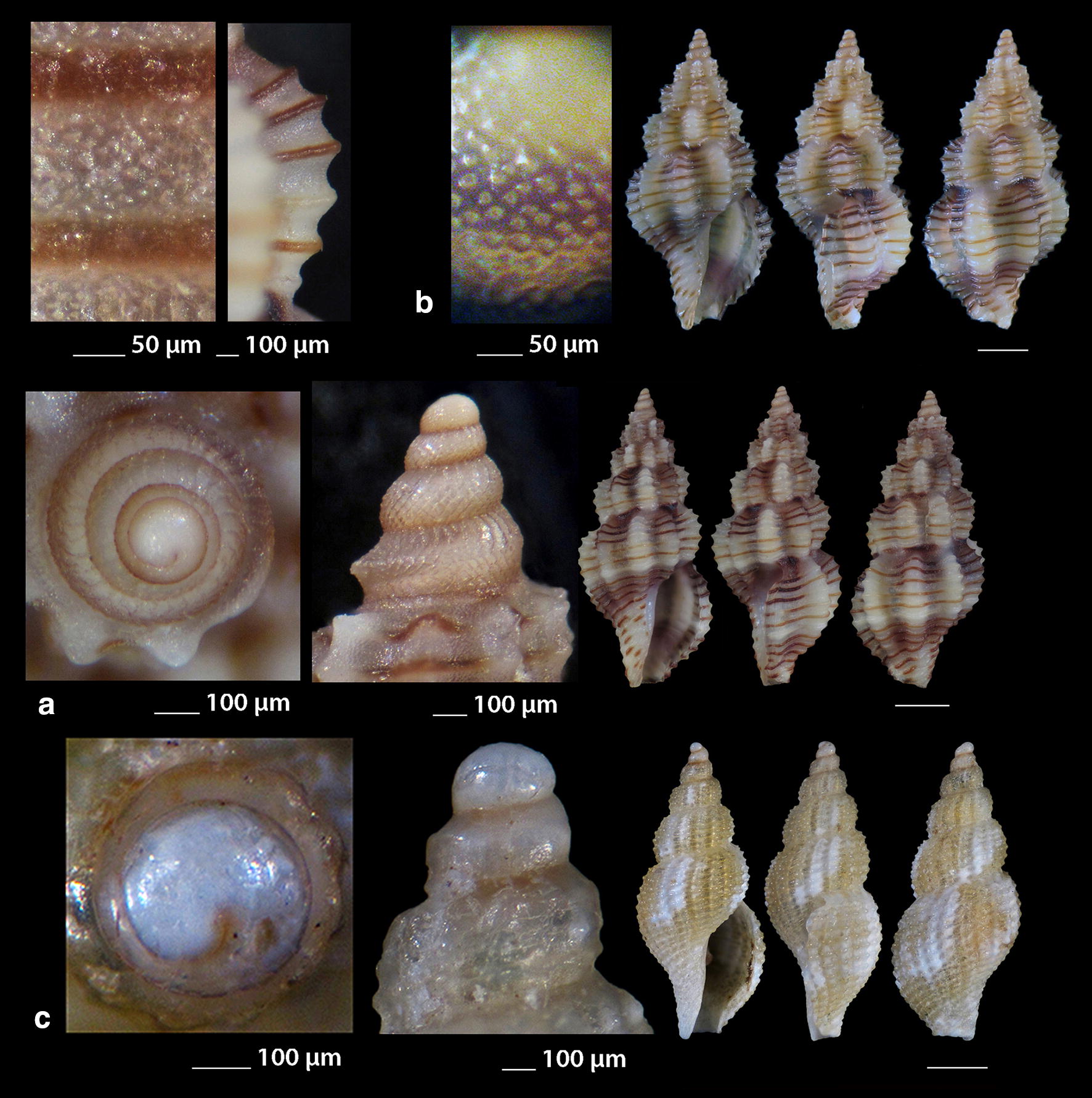
**a**, **b** *Raphitoma aequalis*,** c** *Raphitoma* cf. *alternans.* Bar = 1 mm, unless otherwise indicated

***Raphitoma aequalis*** (Jeffreys, 1867) (Fig. 3a, b)

***Raphitoma*** cf. ***alternans*** (Monterosato, 1884) (Fig. 3c). Due to the very limited literature on *R. alternans* coloration we felt obliged to present our light beige specimen as cf. Given the extensive color variability of other congenerates, we are of the opinion that it will be shown in the future that the species does not only bear irregular white spots on a dark brown background [16].

**Fig. 4:**
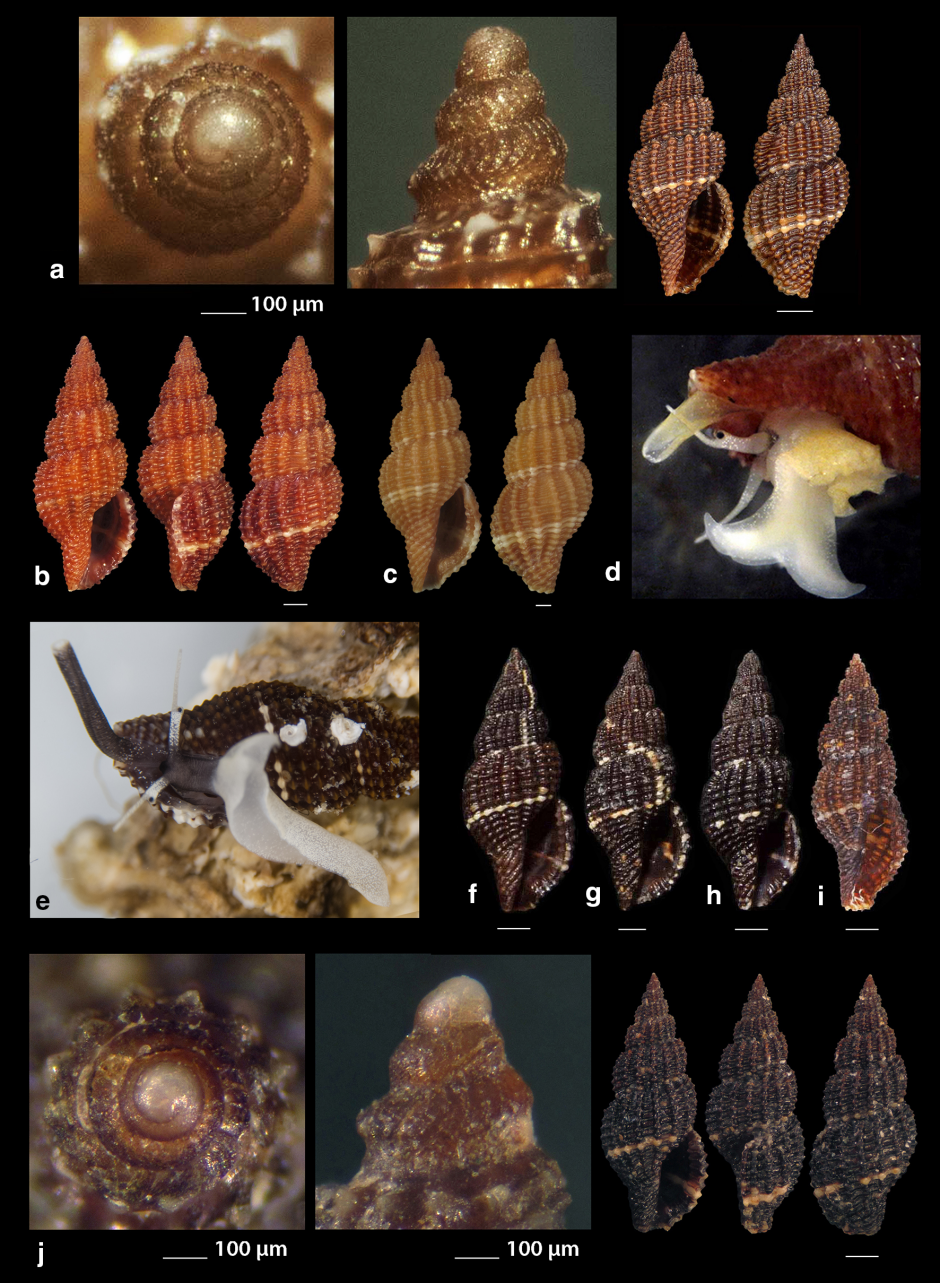
**a**–**j** *Raphitoma atropurpurea*, **a**–**d** deep water form, **d** live specimen of deep water form, **e**–**j** shallow water form, **e** live specimen of shallow water form. Bar = 1 mm, unless otherwise indicated

***Raphitoma atropurpurea*** (Locard & Caziot, 1900) (Fig. 4a–j)

*Collection stations* Six live individuals and thirteen shells (4.20–13.50 mm long, 2.10–4.75 mm wide): − 30 m, mixed bottom, Central Thermaikos Gulf, 40°11′N–24°02′Ε; − 2 m, Korinthiakos Gulf, 38°01′N–22°51′Ε; − 100 m, Central Saronikos Gulf, 37°44′N–23°48′Ε.

*Description* Shell hyaline, robust, fusiform, 2.4–2.6 times as long as wide. Protoconch multispiral, mean 425 μm wide and 470 μm high, of almost 3 diagonally cancellated and convex whorls, the last of which exhibits a weak keel with undulations before the onset of the teleoconch. Teleoconch of 6–7 highly convex whorls separated by a deep suture. The body whorl occupies almost 55% of the total length and bears 14–15 axial ribs with interspaces approximately as wide as the ribs, and 19–21 densely spaced spiral cords much thinner than the ribs, 7 of which are situated above the aperture and the rest 11–15 below the suture. The spiral cords in their intersections with the axial ribs form elongated rectangular tubercles. The tubercles on the first 2 adapical cords are spiny and close to each other forming a narrow subsutural ramp. In fresh specimens the inner wall of the shell viewed through the aperture exhibits a transparency. The aperture occupies approximately 40% of the shells length and exhibits a rough and sigmoid columella, angled at its upper part. Anterior siphonal canal short, well-marked and wide while the posterior one is deep, narrow and pushing the ribs together. The outer lip is thickened internally and bears 10–11 strong teeth with the first one delimiting the posterior canal and the last the anterior. Shell color varies: specimens from mixed bottoms below 50 m of depth (Fig. 4a–d) are uniformly honey-yellow to light orange-tan with an off white band as an extension of the suture on the body whorl, while those of hard substrate above 20 m of depth (Fig. 4e–j) are deep purple-brown with occasional irregularly situated and of limited extension areas of cream-white, an off white subsutural band as an extension of the suture on the body whorl and of lighter color the teeth of the outer lip. Animal color of the deeper form (Fig. 4d) is translucent white with white speckles allover and a gray ring at the base of the tentacles, while the shallower form differs in its dark gray siphon and body (Fig. 4e). Τhe above color variability may be suggestive of two cryptic species.

*Similar species R*. *atropurpurea* superficially resembles *R. contigua* (Monterosato, 1884) but is much slender, with more dense sculptural decoration, a shorter body whorl and aperture, and of different color and color pattern [16, 39].

*Habitat and distribution* Mediterranean Sea [16, 20].

*Status* Uncommon [31]. Second documented record for the Hellenic Seas. Tenekidis [40] reported it as common from Greece misidentified, though, as *R. purpurea* [20].

**Fig. 5:**
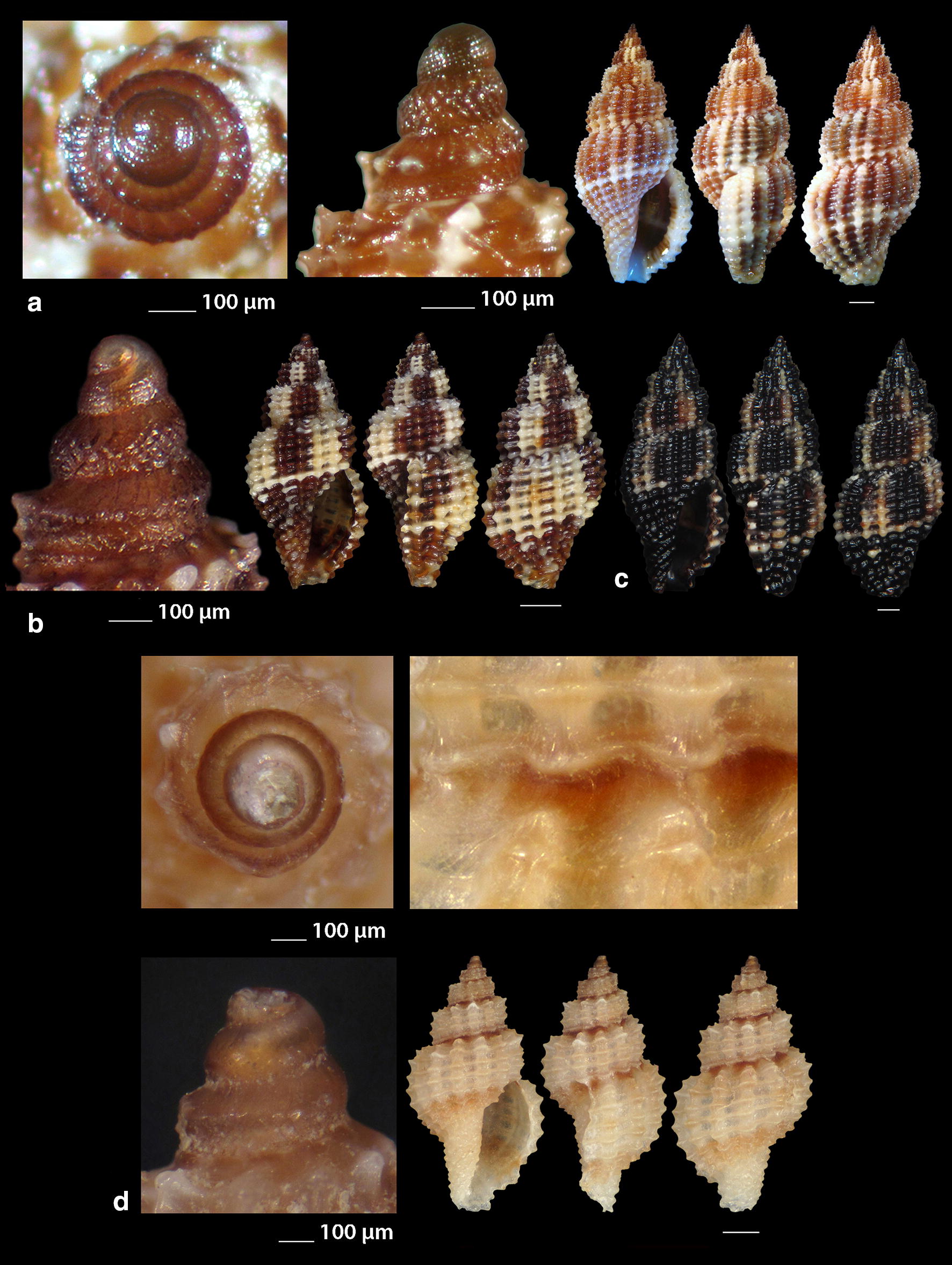
**a**–**c** *Raphitoma bicolor*, **d** *Raphitoma brunneofasciata.* Bar = 1 mm, unless otherwise indicated

***Raphitoma bicolor*** (Risso, 1826) (Fig. 5a–c)

*Collection stations* 24 shells (10.60 mm long, 4.20 mm wide) − 10 m, mixed bottom Palioura, Epanomi, 40°25′N–22°52′Ε; − 8/14 m, mixed bottom, under stones, Anavyssos, Attiki, 37°43′N–23°56′E; Central Saronikos Gulf, 37°45′N–23°48′Ε; Korinthiakos Gulf; Kythnos Island, 37°27′N–24°27′Ε.

*Description* Shell hyaline, robust, fusiform and 2.2 times as long as wide. Multispiral protoconch, mean 420 μm wide and 480 μm high, of 2.65 diagonally cancellated and convex whorls the first of which is decorated with regularly placed small tubercles and its last whorl with a weak keel with erasures before the onset of the teleoconch. The teleoconch consists of 6 convex whorls separated by a deep suture. The body whorl occupies almost 65% of the total length and bears 15–16 slightly opisthocline axial ribs with interspaces approximately 1.5 times wider than the ribs and 17–18 spiral cords slightly thinner than the ribs, 7 of which are situated above the aperture and the rest 11 below the suture. The spiral cords in their intersections with the axial ribs form erasures in the form of small elongated rectangular tubercles. The tubercles on the first 2 adapical cords are spiny and close to each other forming a narrow subsutural ramp. The inner wall of the shell viewed through the aperture exhibits a transparency. The aperture occupies approximately 45% of the shells length and exhibits a smooth and sigmoid columella, angled at its upper part. The anterior siphonal canal is short and wide while the posterior one is deep and narrow. The outer lip bears 11 strong teeth with the first one delimiting the posterior canal and the last the anterior. The color is either uniformly light orange-tan with irregularly situated areas of cream-white and a white subsutural band by the ramp and one more as an extention of the suture on the body whorl or dark purple brown with white rib pairs and a white subsutural band by the ramp and one more as an extention of the suture on the body whorl. Protoconch I chestnut brown and the rest dark orange-tan with white erasures.

*Similar species R*. *bicolor* superficially resembles a number of congeneric Mediterranean *Raphitoma* species but it is different from: *R. alternans* (Monterosato, 1884) in that the late has a paucispiral protoconch, a more elongated shell and a different color pattern; *R. atropurpurea* in its color pattern and in its less slender spire; *R. densa* (Monterosato, 1884) in the shorter body whorl of the late; *R. lineolata* (Bucquoy, Dautzenberg and Dollfus, 1883) in its more inflated profile and its more robust shell; *R*. *contigua*, in its wider aperture and the more inflated body whorl. Due to similarity, it is considered sibling species to the paucispiral protoconch *R. farolita* Nordsieck, 1977 [17].

*Habitat and distribution* Under stones on mixed bottoms. Mediterranean Sea.

*Status* First documented record for the Hellenic Seas.

***Raphitoma brunneofasciata*** Pusateri & Giannuzzi Savlli, 2013

nom. nov. pro *R. brevis* Nordsieck, 1977, non Seguenza, 1880 [17] (Fig. 5d)

*Collection stations* Five immature shells (4.30–6.90 mm long, 2.40–3.70 mm wide) − 60 m, mixed bottom, Central Saronikos Gulf, 37°54′N–23°39′E; − 3 m, mixed bottom, Pantokratoras, Preveza, 38°94′N–20°73′E.

*Description* Shell hyaline, biconic, fusiform and 1.8 times as long as wide. Its multispiral protoconch has its nucleus chipped off, is approximately 440 μm wide and consists of almost 2.60 diagonally cancellated and convex whorls the last of which with a weak keel before the onset of the teleoconch. The teleoconch consists of 4 convex whorls separated by a deep suture and exhibiting a conspicuously strong shoulder. The body whorl occupies almost 70% of the total length and bears 12–13 orthocline axial ribs with interspaces approximately equal in width to the ribs and 17 thin spiral cords, 5 of which are situated above the aperture and the rest 12 bellow. The spiral cords in their intersections with the axial ribs form erasures in the form of small elongated rectangular tubercles. The tubercles on the first 2 adapical cords are spiny forming a strong subsutural ramp. The inner wall of the shell viewed through the aperture exhibits a transparency. Aperture wide, occupies approximately 50% of the shell length and exhibits a rough and sigmoid columella, angled at its upper part. The anterior siphonal canal is long and wide while the posterior one is short. Color uniformly cream-white with a light brown subsutural band by the ramp and a wider one as an extention of the suture on the body whorl. Protoconch also light brown. Teleoconch covered all over its surface with small erasures.

*Similar species R*. *brunneofasciata* superficially resembles *R. echinata* (Brocchi, 1814) in its spicky outlook, but is much more inflated than the latter and is covered all over its surface with small erasures.

*Habitat and distribution* Mixed bottom with *Posidonia oceanica*. Known from the Ibiza region [39].

*Status* Second documented record for the Hellenic Seas [41].

**Fig. 6:**
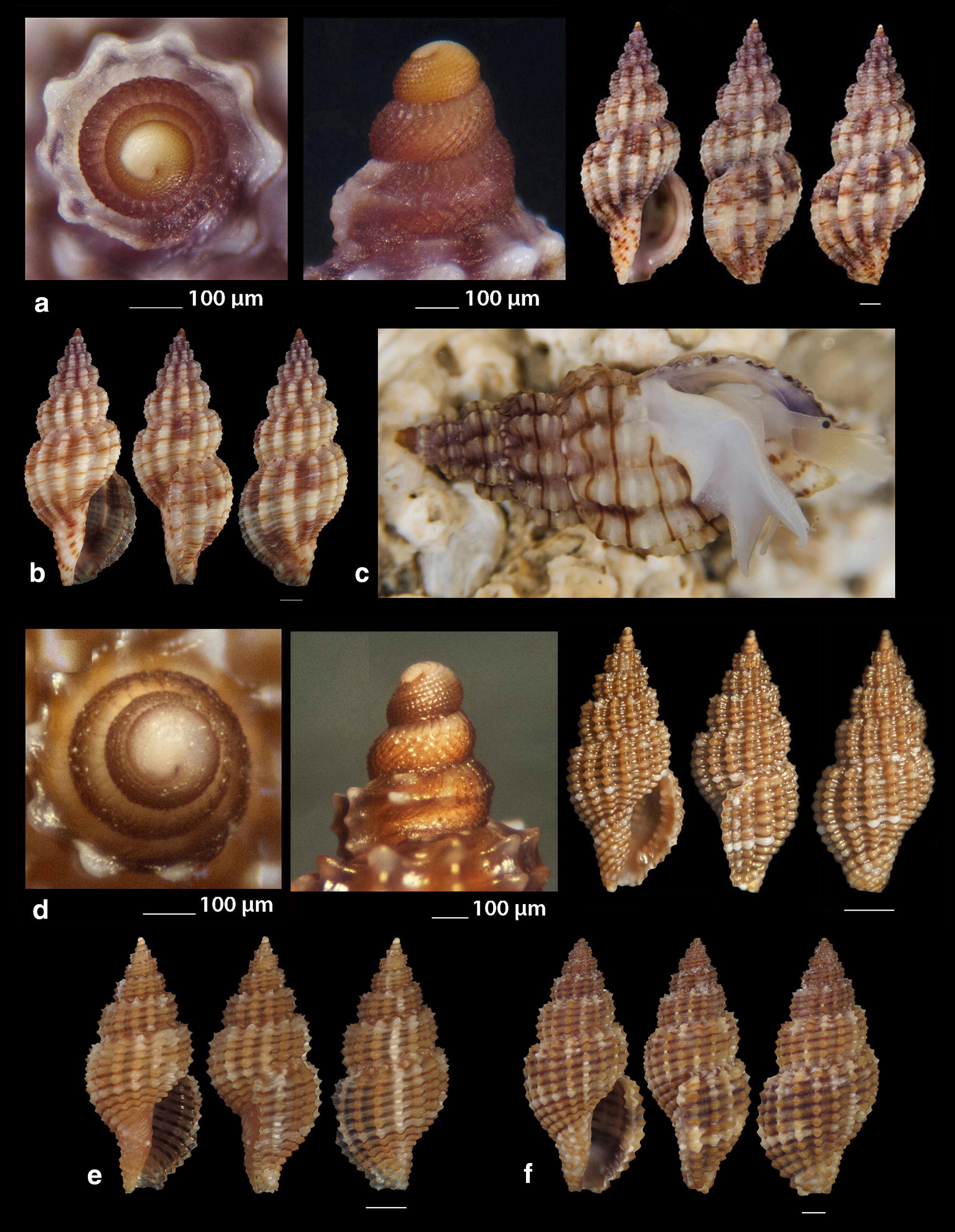
**a**–**c** *Raphitoma concinna*, **c** live specimen, **d**–**f** *Raphitoma contigua*. Bar = 1 mm, unless otherwise indicated

**Fig. 7:**
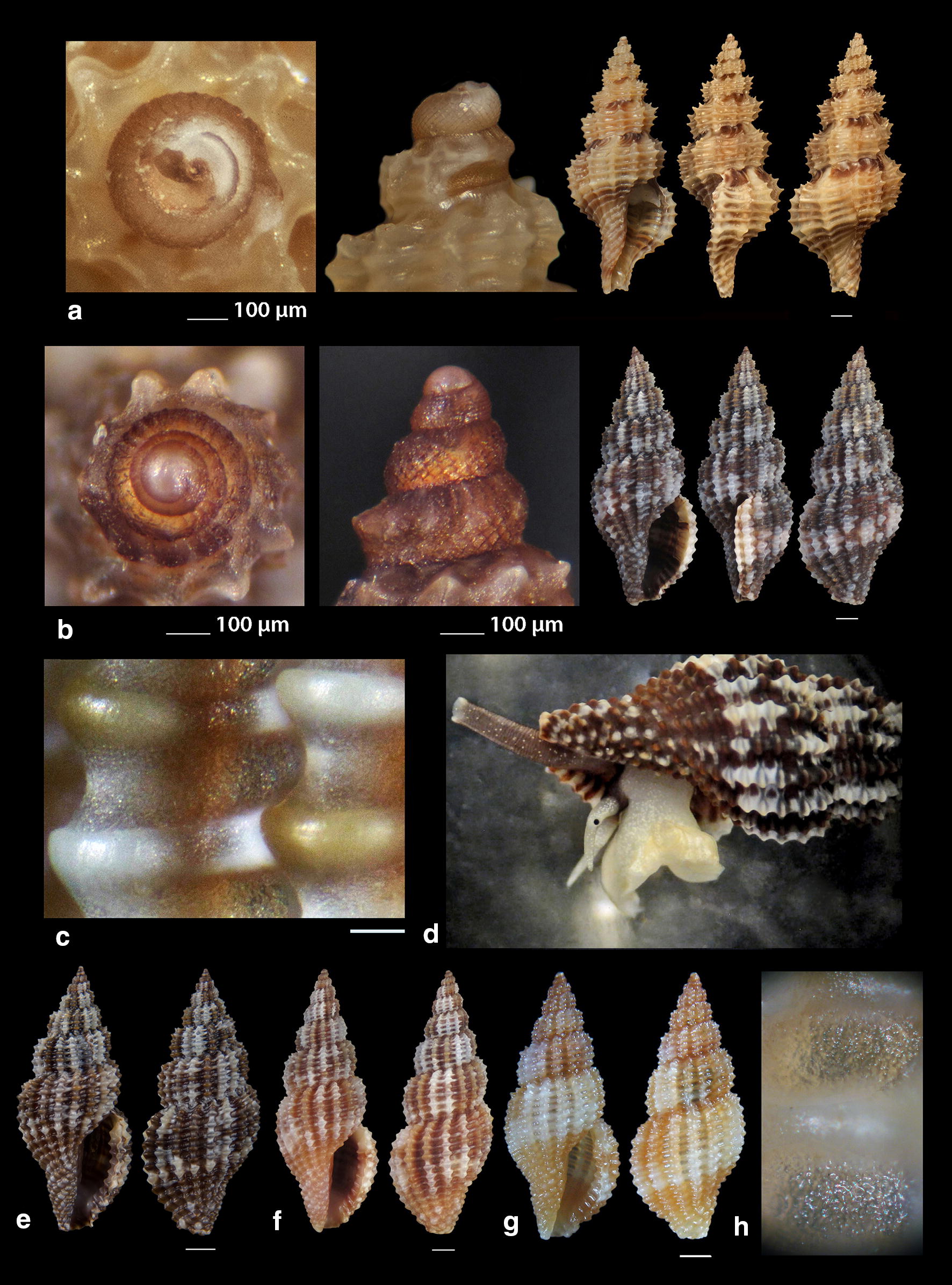
**a** *Raphitoma cordieri*, **b**–**h**. *Raphitoma densa*, **c** surface of specimen (**b**), **d** live specimen, **h** surface of specimen (**g**). Barr = 1 mm, unless otherwise indicated

***Raphitoma concinna*** (Scacchi, 1836) (Fig. 6a–c), ***Raphitoma contigua*** (Monterosato, 1884) (Fig. 6d–f), ***Raphitoma cordieri*** (sensu Cossignani & Ardovini, 2011) (Fig. 7a), ***Raphitoma densa*** (Monterosato, 1884) (Fig. 7b–h)

**Fig. 8:**
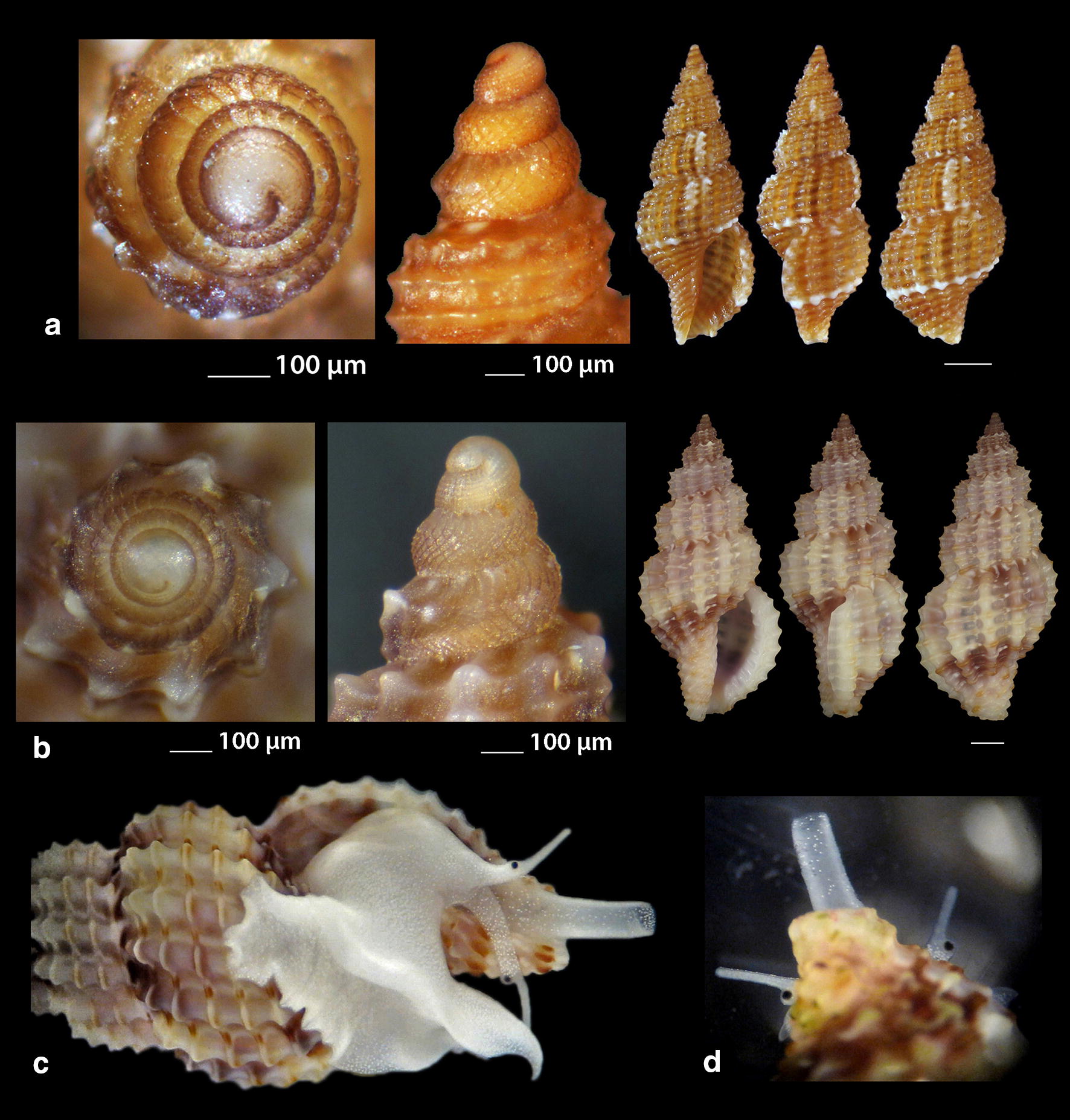
**a** *Raphitoma digiulioi*, **b**–**d** *Raphitoma echinata* (sensu *auctores*) Morphotype I, **c**, **d** live specimens. Bar = 1 mm, unless otherwise indicated

***Raphitoma digiulioi*** Pusateri & Giannuzzi Savelli 2017 (Fig. 8a)

*Collection station* One shell (6.45 mm long, 2.55 mm wide) − 80 m, mixed bottom, Pyrgadikia, Chalkidiki, 40°18′N–23°45′Ε.

*Description* Shell smooth, hyaline, fusiform, acute and 2.5 times as long as wide. Protoconch multispiral, 390 μm wide, 460 μm high. Protoconch I of 0.7 whorls, 185 μm wide with orthogonally cancellated sculpture. Protoconch II of 2.25 diagonally cancellated and convex whorls the last of which exhibits a keel with undulations before the onset of the teleoconch. Protoconch–teleoconch sinusoidal boundary. The teleoconch consists of 5.5 convex whorls with a weak ramp by a deep suture. The body whorl occupies almost 55% of the total length and bears 16–17 orthocline to opisthocline axial ribs approximately as wide as the interspaces, and 17 densely spaced spiral cords slightly thinner than the ribs, 5 of which are situated above the aperture and the rest below. The spiral cords in their intersections with the axial ribs form rectangular tubercles. Τhe inner wall of the shell viewed through the aperture exhibits a transparency. The aperture occupies approximately 40% of the shell length and exhibits a simple and slightly sigmoid columella, angled at its upper part. Anterior siphonal canal short and wide, posterior deep and narrow. Outer lip thickened internally with 10 weak denticles. Shell color honey-yellow with randomly placed white spots on the whorls, sickle-shaped spots on the ramp by the suture and a white cord as an extention of the suture on the body whorl.

*Similar species R. digiulioi* is slender than *R. atropurpurea*, with more axial ribs and spiral cords above the aperture, weaker sculpture, and smaller protoconch (heigth 450 μm, width 390 μm vs heigth 485 μm, width 420 μm) [20]. It is quite dissimilar to *R. lineolata* (Bucquoy, Dollfus & Dautzenberg, 1883) as the latter has a smaller protoconch (height 315 µm, width 325 µm vs heigth 450 μm, width 390 μm) [17], protoconch I of 1.2 whorls (instead of 0.7), protoconch II of 1.5 whorls (instead of 2.25) and more spiral cords above the suture (8 vs 5–6) [17, 20]. It is also smaller than *R. purpurea* (Montagu, 1803), has a less robust shell, a more acute teleoconch, stronger axial ribs and tubercles, lacks the microgranules on the teleoconch, and bears a smaller protoconch (heigth 450 μm, width 390 μm vs height 640 μm, width 460 μm) [20].

*Habitat and distribution* Central Mediterranean Sea [20].

*Status* First documented record for the Hellenic Seas and the East Mediterranean Sea.

**Fig. 9:**
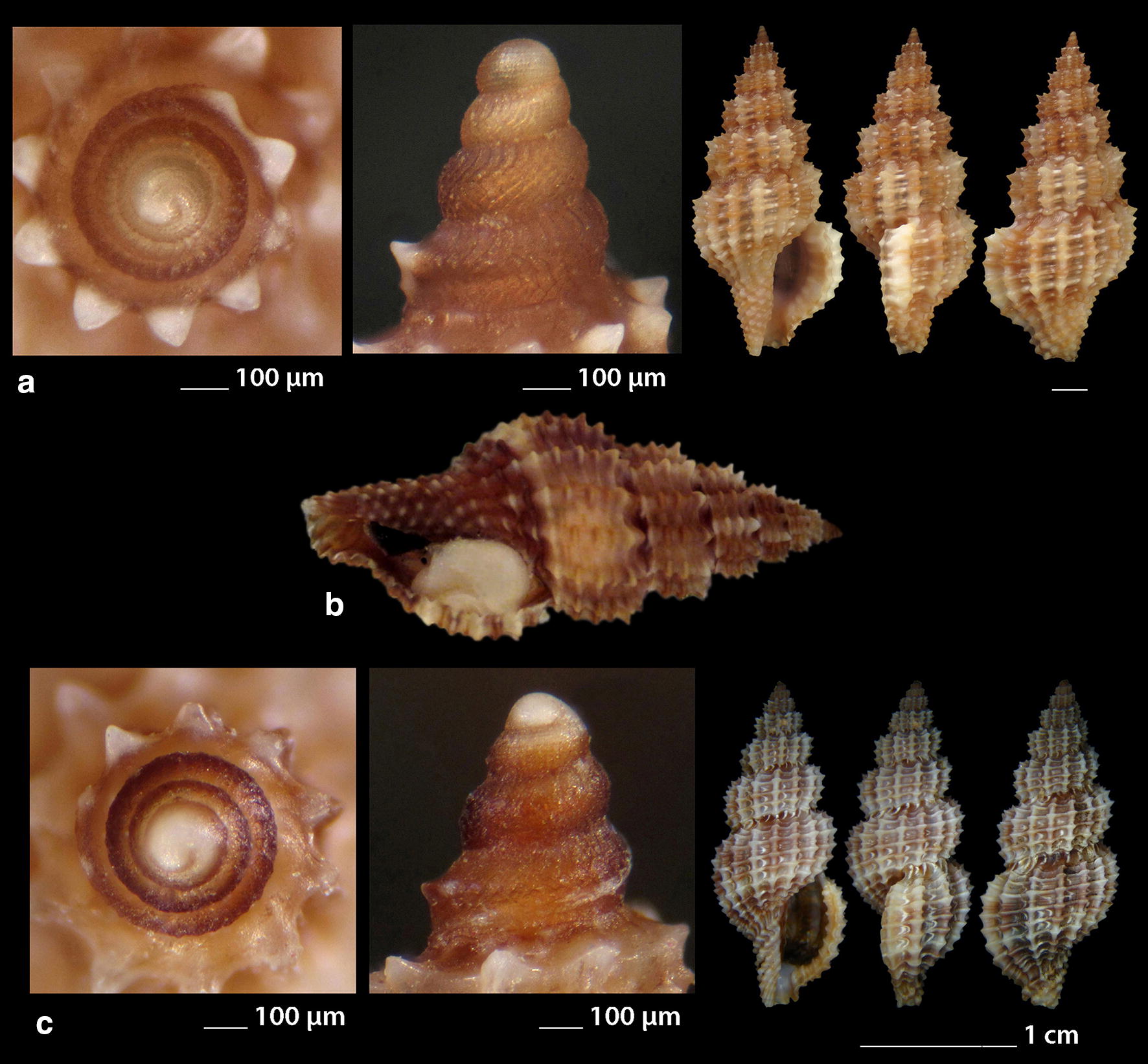
**a**, **b** *Raphitoma echinata* (sensu *auctores*) Morphotype II, **b** live specimen, **c** *Raphitoma echinata* (sensu *auctores*) Morphotype III. Bar = 1 mm, unless otherwise indicated

***Raphitoma echinata*** (sensu *auctores*) Morphotype I (Fig. 8b–d), ***Raphitoma echinata*** (sensu *auctores*) Morphotype II (Fig. 9a, b), ***Raphitoma echinata*** (sensu *auctores*) Morphotype III (Fig. 9c)

**Fig. 10:**
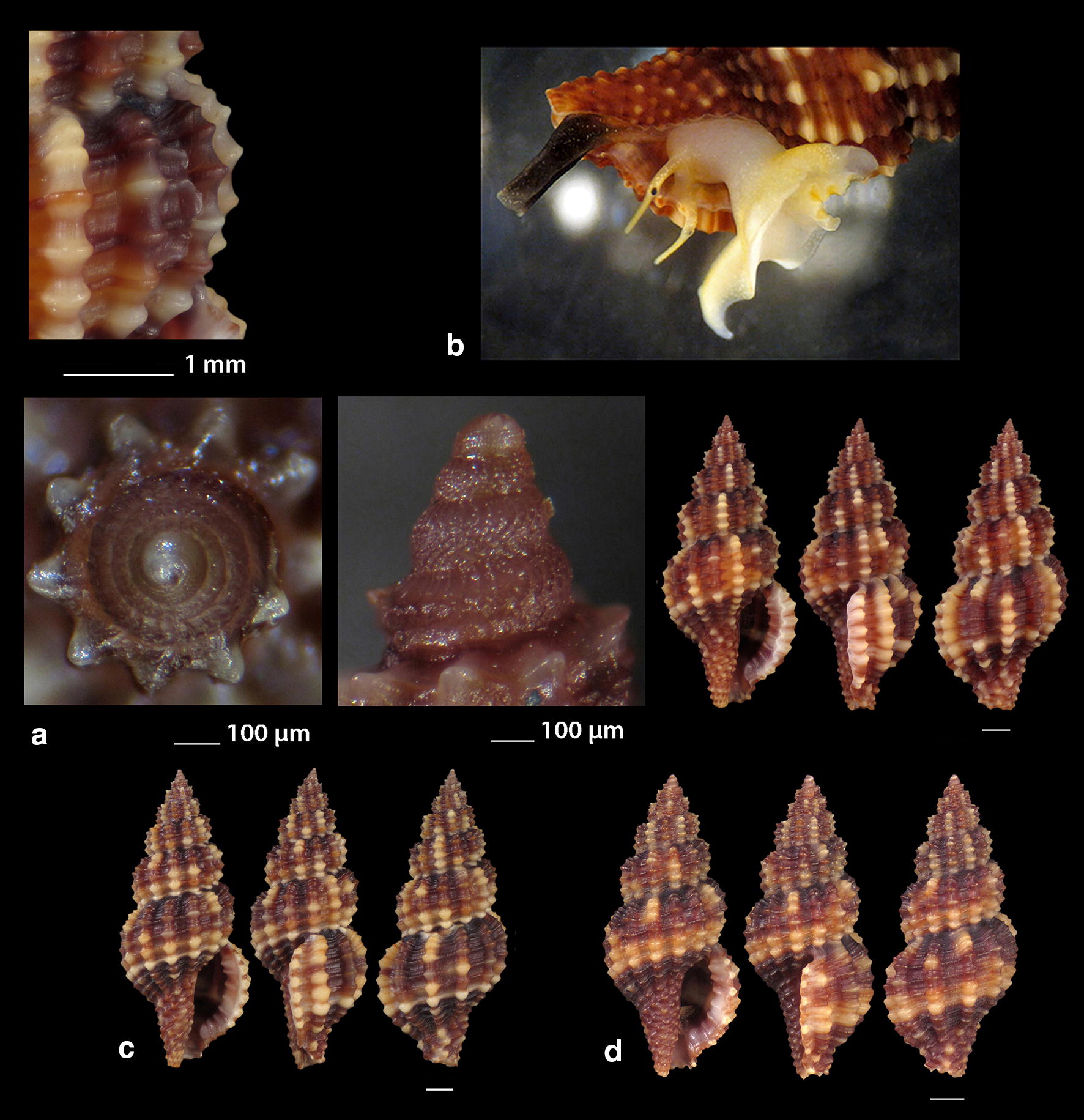
**a**–**d** *Raphitoma ephesina*, **b** live specimen. Bar = 1 mm, unless otherwise indicated

***Raphitoma ephesina*** Pusateri, Giannuzzi Savelli & Stahlschmidt, 2017 (Fig. 10a–d)

*Collection stations* Eight live specimens and twelve shells (6.10 mm to 11.00 mm long, 3.20–4.50 mm wide), − 2/100 m, mixed bottoms and maerl, East Korinthiakos Gulf, 38°17′N–22°34′E; West Korinthiakos Gulf, 38°01′N–22°51′Ε, Central Saronikos Gulf, 37°54′N–23°39′E.

*Description* Shell biconic, porcelaneous, robust, inflated and nearly 2.25 times as long as wide. Multispiral protoconch, mean 420 μm wide and 570 μm high, nearly 3.5 diagonally cancellated and convex whorls the first of which is decorated with regularly placed small tubercles and its last whorl with a weak keel before the onset of the teleoconch. The latter consists of 6 highly convex whorls separated by a deep suture. Body whorl nearly 60%, 14 slightly prosocline axial ribs with interspaces as wide as the ribs and 15–16 spiral cords much thinner than the ribs, 5 of which are situated above the suture and the rest bellow up to the shell’s short tail. The spiral cords in their intersections with the axial ribs form large mammiliform tubercles that endow the shell with an “ear of maize with regular rows of kernels” appearance. The tubercles on the first 2 adapical cords are vestigial and close to each other forming a very narrow subsutural ramp. Aperture approximately 44% exhibiting a smooth and sigmoid columella angled at its upper part. Anterior siphonal canal strait and slightly elongated and wide, posterior one deep and narrow. Outer lip particularly strong bearing 9 very strong teeth with the first one delimiting the posterior canal and the last the anterior. Color, irregularly chocolate-brown to brown-purple with irregularly situated areas of cream-white, a white band as an extention of the suture on the body whorl and some randomly placed white tubercles. Embryonic shell whitish and larval shell lilac. Animal color yellowish white with white spotlets on the tentacles and on the greyish-black siphon.

*Similar species R. ephesina* seems to belong to the *R. linearis* (Montagu, 1803)—*R. aequalis* (Jeffreys, 1867) group as is that described by Høisæter [42] as it is of a similar profile and size, bears conspicuous spiral cords with colored tops, has the same number of protoconch whorls and almost the same protoconch dimensions. In particular, it differs from *R. linearis* in the absence of microgranules, the number of the spiral cords on the penultimate whorl (5 instead of 4) and the barely noticeable apertural denticles of *R. linearis*. It differs from *R. aequalis* in the absence of micro-granules and in the number of spiral cords on the penultimate whorl (5 instead of 6–7). It could be mistaken for *R. bicolor* (Risso, 1826) juveniles but their protoconches are different as there is a keel present in that of *R. bicolor* [19], (Figs. 12, 13, 14, 15).

*Habitat and distribution* According to Pusateri et al. [19] the species has been initially reported as *R. bicolor* (Risso, 1826) by Peter Stahlschmidt from Bozcaada Island, Turkey and subsequently found in Evia and Saronikos Bay (Greece), the Ligurian Sea (Italy), the Adriatic Sea (Croatia) and the Algerian Basin (Sardinia, Italy).

*Status* Second record for the Hellenic Seas [19].

**Fig. 11:**
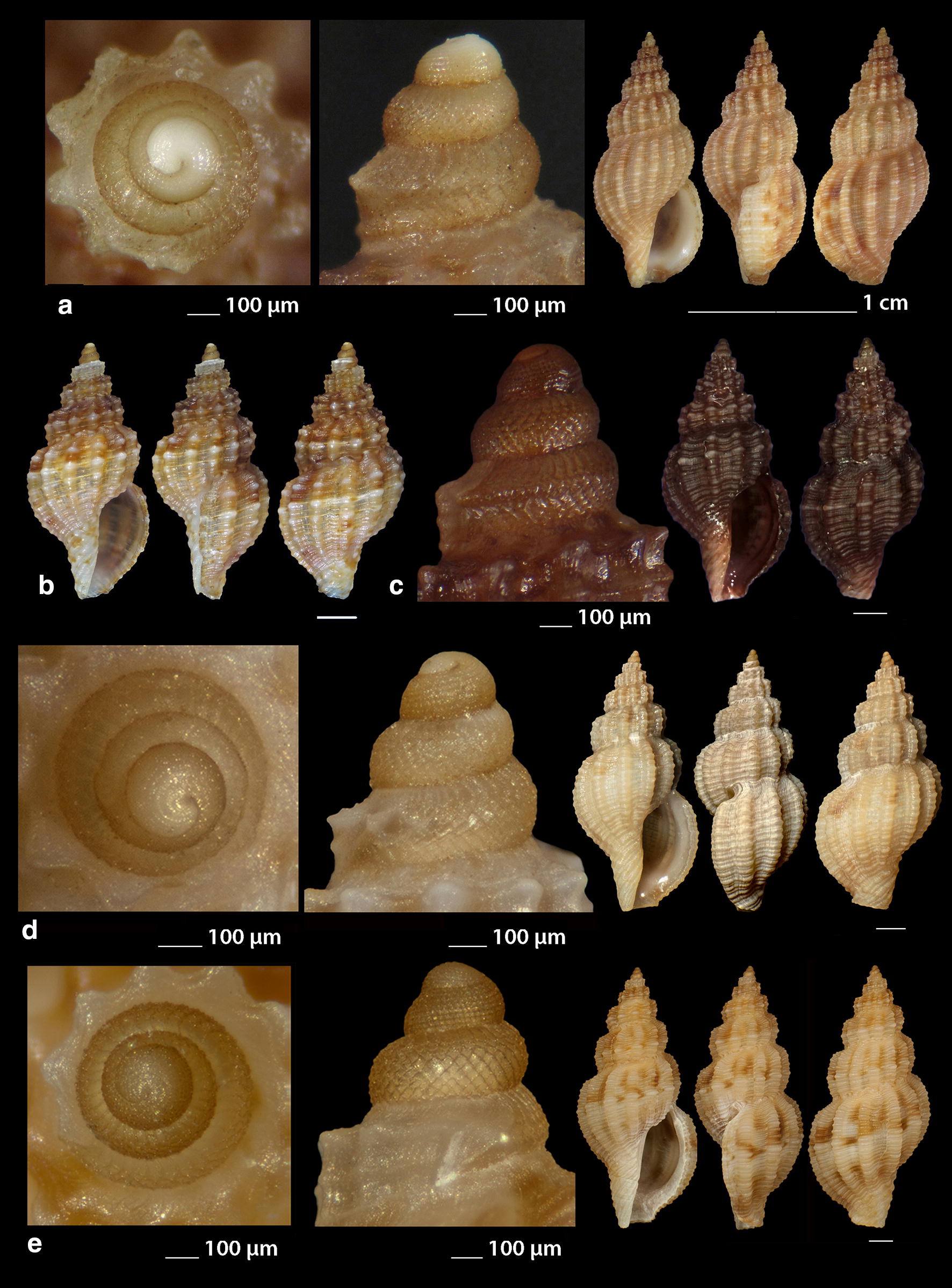
**a**–**e** *Raphitoma erronea*. Bar = 1 mm, unless otherwise indicated

***Raphitoma erronea*** (Monterosato, 1884) (Fig. 11a–e)

*Collection stations* Fourty shells (3.90–15.25 mm long, 2.50–6.55 mm wide), − 60 m, Central Siggitikos Gulf, 40°05′N–22°56′Ε; − 400 m, Lemnos Island, 40°05′N–25°12′E; − 100/120 m, coralligenous substrate, Central Saronikos Gulf, 37°44′N–23°48′Ε; Kythnos Island, 37°27′N–24°27′Ε; − 100/150 m, coral debris, Korintiakos Gulf, 38°19′N–22°19′Ε; − 60 m, Kardamili, Messinia, 36°51′N–22°13′E.

*Description* Shell biconic, elongated, scalariform and nearly 2.3 times as long as wide. Blunt and multispiral protoconch, mean 560 μm wide and 640 μm high, of 2.75 highly cancellated and convex whorls. The cancellation starts straight from the nucleus while the last whorl exhibits a weak keel before the onset of the protoconch. Protoconch I of mean 1.2 whorls and mean 310 μm in diameter. Teleoconch with 6.0 convex whorls exhibiting a slight shoulder immediately below the deep suture. Body whorl almost 60% and bears 15–16 orthocline axial ribs with interspaces approximately as wide as the ribs. Spiral decoration with cords some wider of which alternating with more numerous thinner ones and forming ambient tubercles at the crossings with the ribs. Aperture slightly more than 40%, elongated and with a short tail. Columella smooth and sinuous. Outer lip simple and thickened, anterior siphonal canal short and wide, posterior one deep and wide. Background color beige or yellowish to chocolate-brown turning lighter towards the body whorl, with darker smudges arranged in an irregular manner, with a whitish spiral band on the middle of the spire, of lighter color the tubercles at the crossings between the ribs and the cords, while the protoconch is either light beige or lemon-yellow. Protoconch nucleus whitish.

*Similar species* Some species of the genus *Raphitoma*, such as *R. concinna* (Scacchi, 1836) and *R. leufroyi* (Michaud, 1828), are very similar to *R. erronea* but, apart from having a protoconch with more whorls, they also have a different sculpture of the teleoconch. It is also often erroneously identified as *Pleurotomella demosia* (Dautzenberg & Fischer, 1896) but the latter has 1 whorl less on its protoconch and a longer tale of the teleoconch [43–45].

*Habitat and distribution* Coralligenous infralittoral bottoms of the West and Central Mediterranean Sea [31, 45, 46].

*Status* Uncommon [31]. First documented record for the Hellenic Seas and the East Mediterranean Sea.

**Fig. 12:**
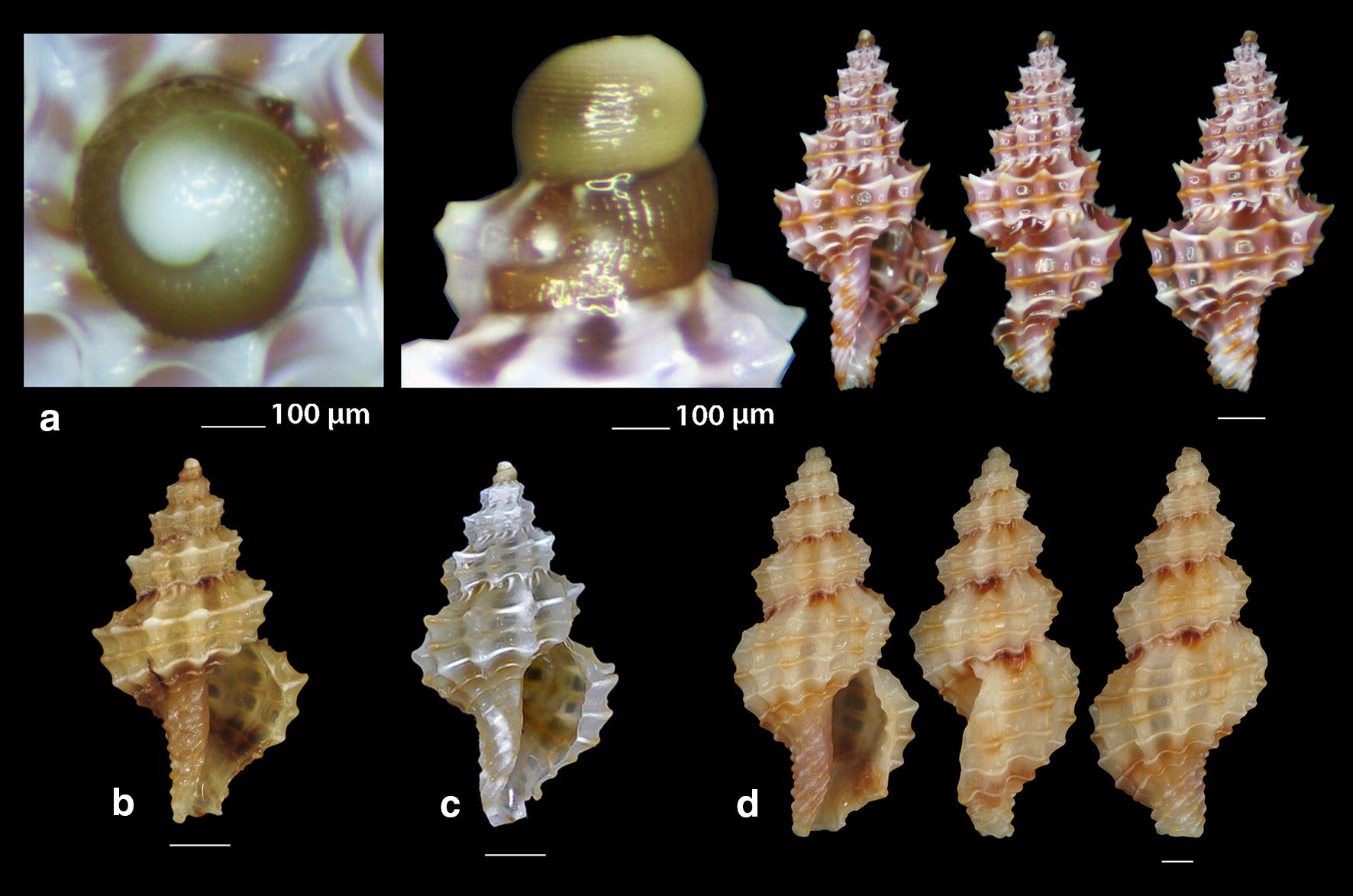
**a**–**d** *Raphitoma horrida*. Bar = 1 mm, unless otherwise indicated

***Raphitoma horrida*** (Monterosato, 1884) (Fig. 12a–d)

**Fig. 13:**
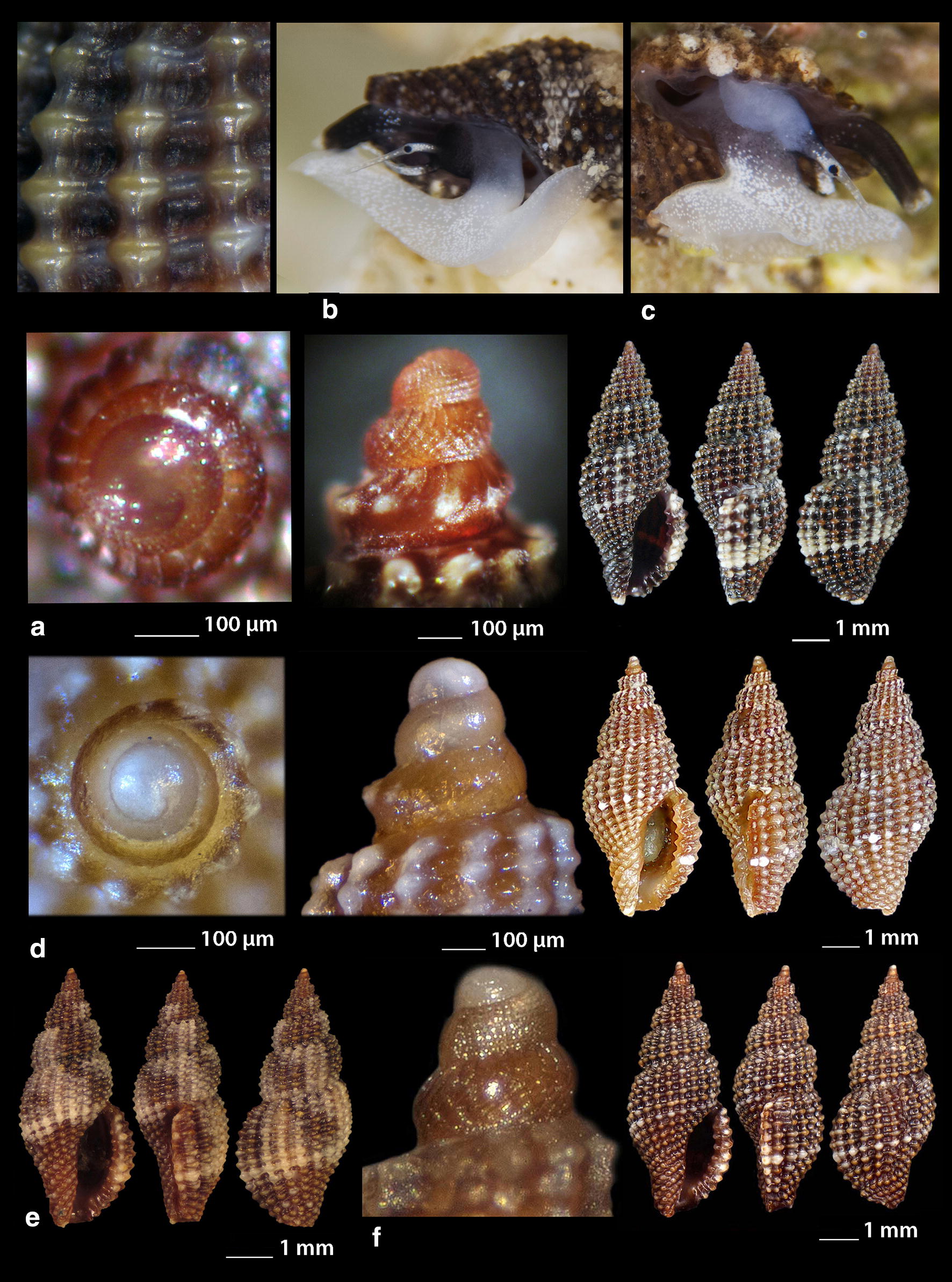
**a**–**f** *Raphitoma laviae*, **b**, **c** live specimens. Bar = 1 mm, unless otherwise indicated

***Raphitoma laviae*** (Philippi, 1844) (Fig. 13a–f)

*Collection stations* Six live individuals and fifty shells (3.50–7.25 mm long, 1.35–2.90 mm wide), − 70 m, mixed bottom, Cape, Epanomi, 40°23′N–22°53′Ε; − 2/120 m, mixed bottoms, Central Thermaikos Gulf, 40°05′N–22°56′Ε; Eandio, Salamina Island, 37°55′N–23°27′E; Lagonissi, Attiki, 37°47′N–23°52′E; Anavyssos, Attiki, 37°43′N–23°56′E; Legrena, Attiki, 37°41′N–23°59′E; Central Saronikos Gulf, 37°54′N–23°39′E; − 50 m, muddy bottom, Lemnos Island, 40°05′N–25°12′E; − 5 m, Pantocratoras, Preveza, 38°94′N–20°73′E.

*Description* Shell hyaline, transparent through the aperture, fusiform, 2.2–2.4 times as long as wide. Multispiral protoconch, mean 380 μm wide and 420 μm high, of 2.3 diagonally cancellated and convex whorls the first of which is decorated with irregularly placed small tubercles and its last whorl with a weak keel before the onset of the protoconch. Teleoconch of 4 slightly convex whorls separated by a deep conspicuous suture. Body whorl occupies 70% of the total length, is inflated and bears 19–20 orthocline to slightly opisthocline axial ribs with interspaces of equal breadth as the ribs. Spiral decoration of 16–17 spiral cords slightly thinner than the ribs, 6 of which are situated above the aperture and the rest below the suture on the body whorl. The spiral cords in their intersections with the axial ribs form erasures in the form of small rounded rectangular tubercles while the interspaces form deep and rounded depressions. The tubercles on the first adapical cord are weaker than the rest thus forming no shoulder. The aperture occupies some 48% of the shells length, has parallel borders and exhibits a smooth and S-shaped columella, angled at its upper part. The anterior siphonal canal is short and distinctively wide while the posterior one is deep and narrow. The particularly robust outer lip bears 9 strong teeth with the first one delimiting the posterior canal and the last the anterior. Some shells are of uniform chestnut-red to orange-red color with lighter color or white the first 2 whorls of the protoconch and the erasures on the intersections between the ribs and the cords, while some others, especially those from deeper waters, are darker to chestnut brown. Irregularly situated single spots or limited lines of cream-white are present on certain shells.

*Similar species R*. *laviae* superficially resembles a number of congeneric Mediterranean *Raphitoma* species but it is different from: *R. alternans* (Monterosato, 1884) in that the late has a paucispiral protoconch, a more elongated shell and a different color pattern; *R. atropurpurea* in its color which is purple-brown in *R. atropurpurea*, in its less slender spire and its smaller size than *R. atropurpurea*; *R. densa* (Monterosato, 1884) in the color and the paucispiral protoconch of the late; *R. lineolata* (Bucquoy, Dautzenberg and Dollfus, 1883) in that *R*. *laviae* has a more inflated profile and a more robust shell; *R. oblonga* (Jeffreys, 1867) in its wider aperture and the different color pattern; *R. spadiana* Pusateri & Giannuzzi-Savelli, 2012 mainly because the late bears a paucispiral protoconch [16].

*Habitat and distribution* West and Central Mediterranean Sea [31, 33].

*Status* Common [39], uncommon [31]. First documented record for the Hellenic seas.

**Fig. 14:**
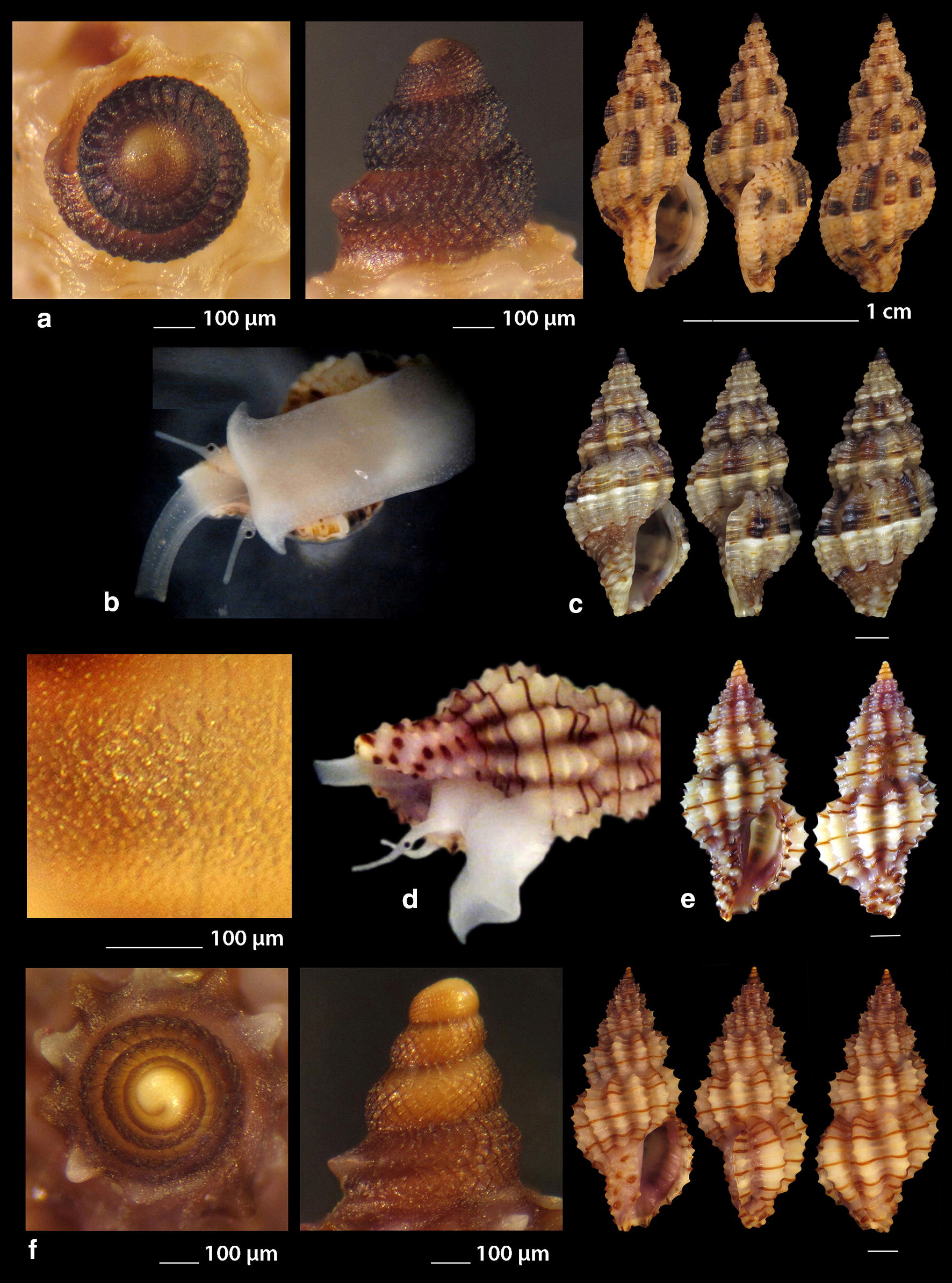
**a**–**c** *Raphitoma leufroyi*, **b** live specimen, **d**–**f** *Raphitoma linearis*, **d** live specimen. Bar = 1 mm, unless otherwise indicated

**Fig. 15:**
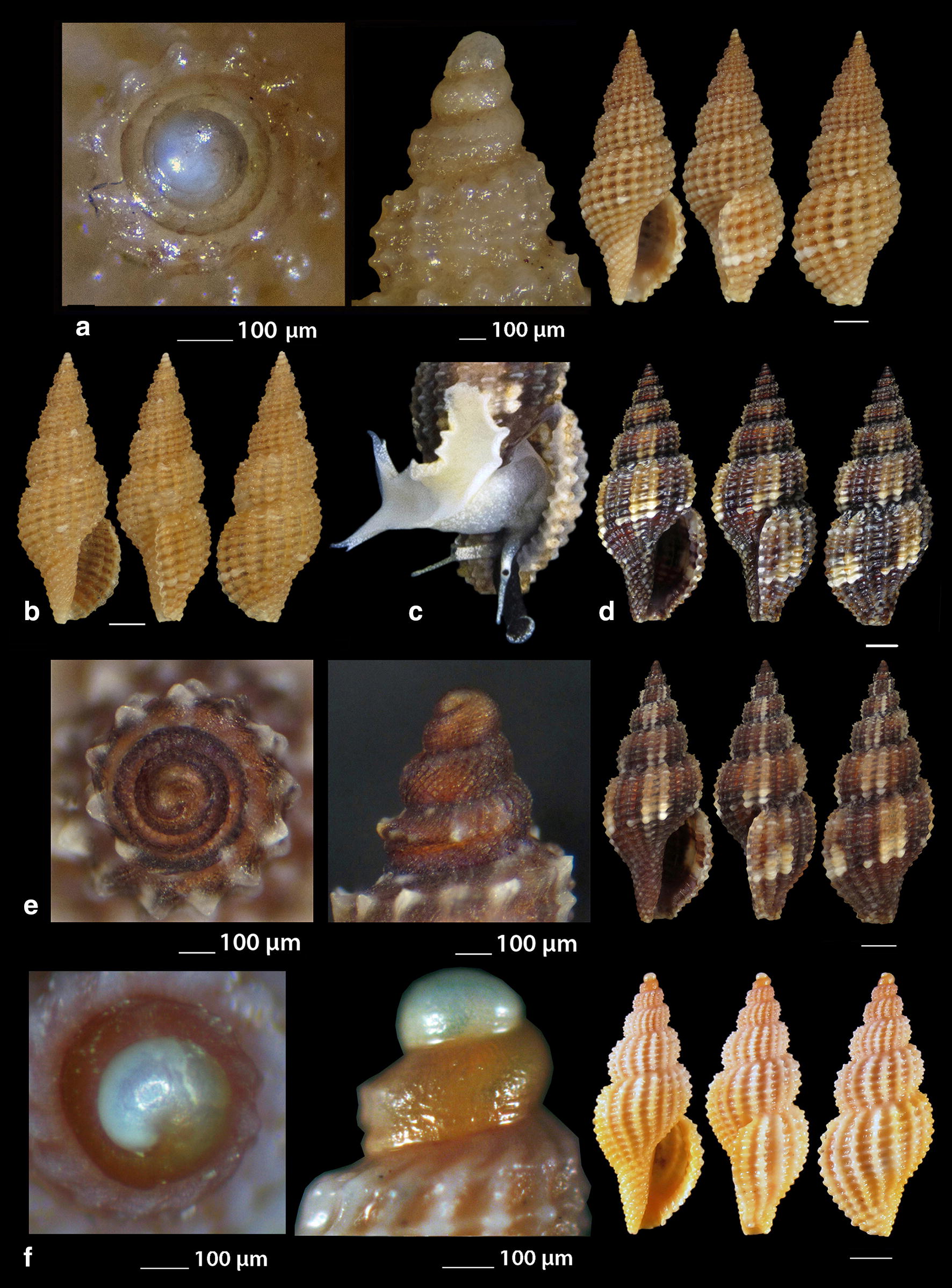
**a**, **b** *Raphitoma lineolata*. **c**–**e** *Raphitoma locardi*, **c** live specimen. **f** *Raphitoma papillosa*. Bar = 1 mm, unless otherwise indicated

**Fig. 16:**
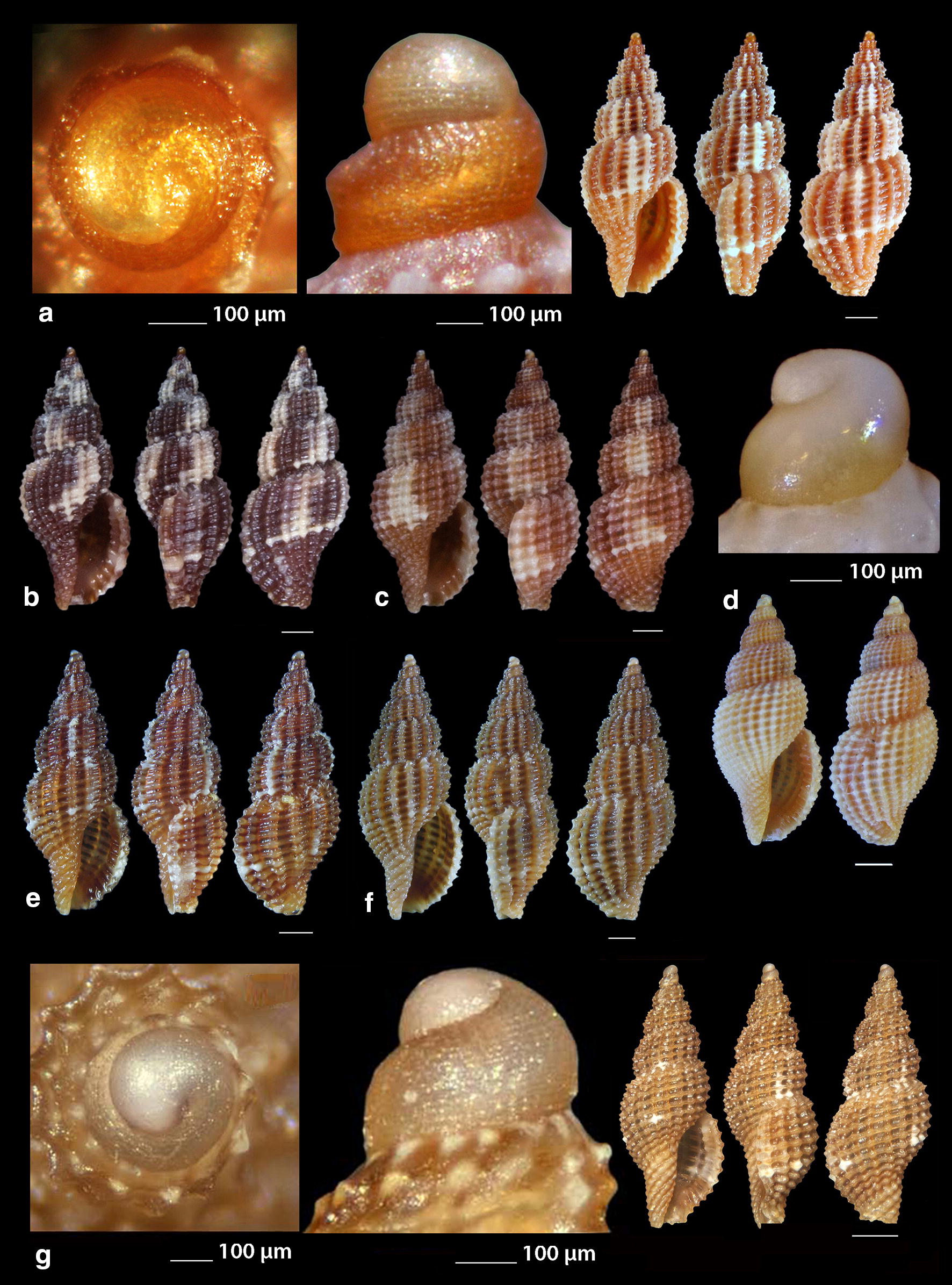
**a**–**c** *Raphitoma philberti*, **d** *Raphitoma pruinosa*, **e**–**g** *Raphitoma smriglioi.* Bar = 1 mm, unless otherwise indicated

**Fig. 17:**
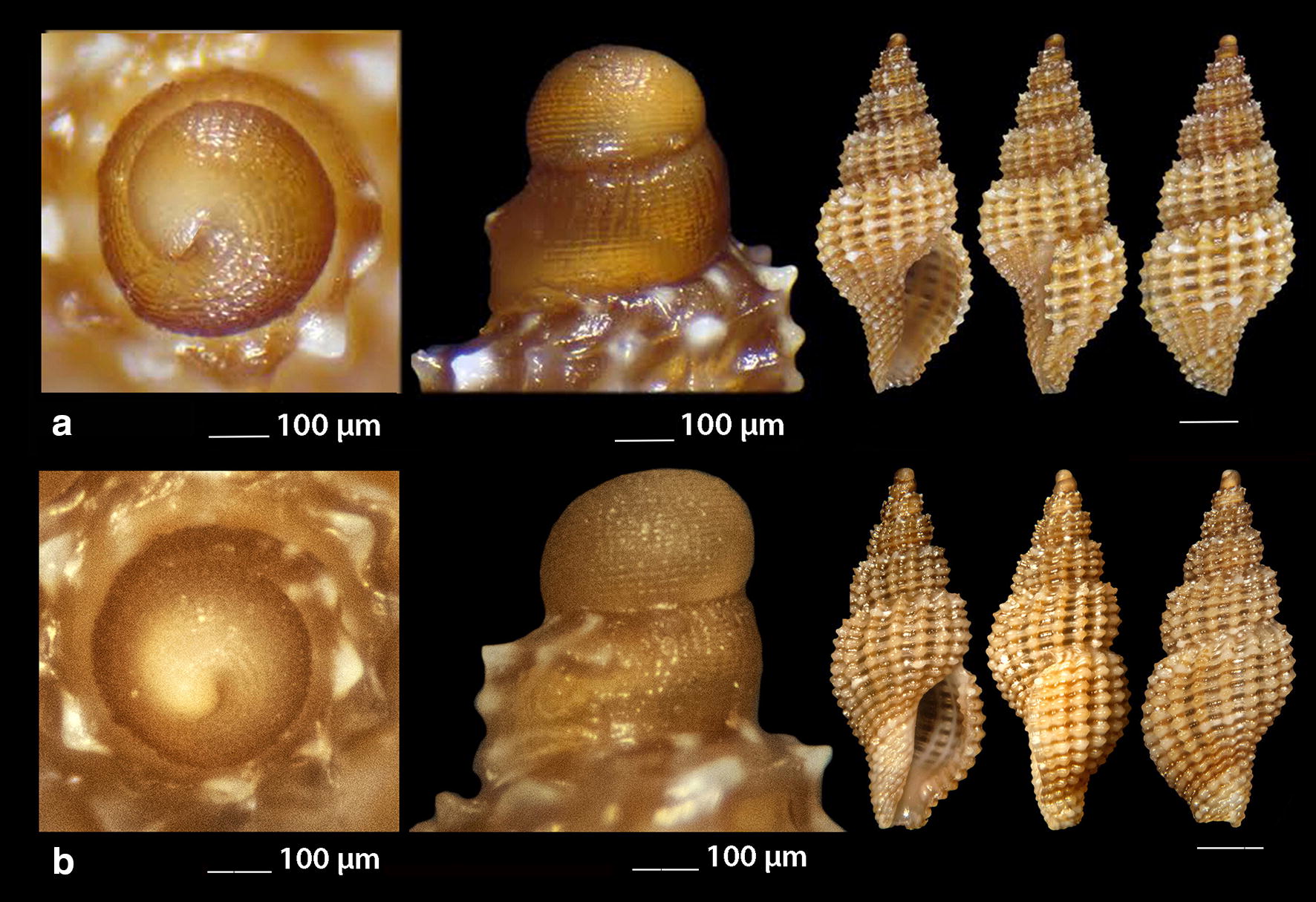
**a**, **b** *Raphitoma spadiana*. Bar = 1 mm, unless otherwise indicated

***Raphitoma leufroyi*** (Michaud, 1828) (Fig. 14a–c), ***Raphitoma linearis*** (Montagu, 1803) (Fig. 14d–f) ***Raphitoma lineolata*** (Bucquoy, Dollfus & Dautzenberg, 1883) (Fig. 15a, b), ***Raphitoma locardi*** Pusateri & Giannuzzi 2013 (Fig. 15c–e), ***Raphitoma papillosa*** (Pallary, 1904) (Fig. 15f), ***Raphitoma philberti*** (Michaud, 1829) (Fig. 16a–c), ***Raphitoma pruinosa*** (Pallary, 1906) (Fig. 16d), ***Raphitoma smriglioi*** Pusateri & Giannuzzi-Savelli, 2013 (Fig. 16e–g), ***Raphitoma spadiana*** Pusateri & Giannuzzi-Savelli, 2012 (Fig. 17a, b).

**Fig. 18:**
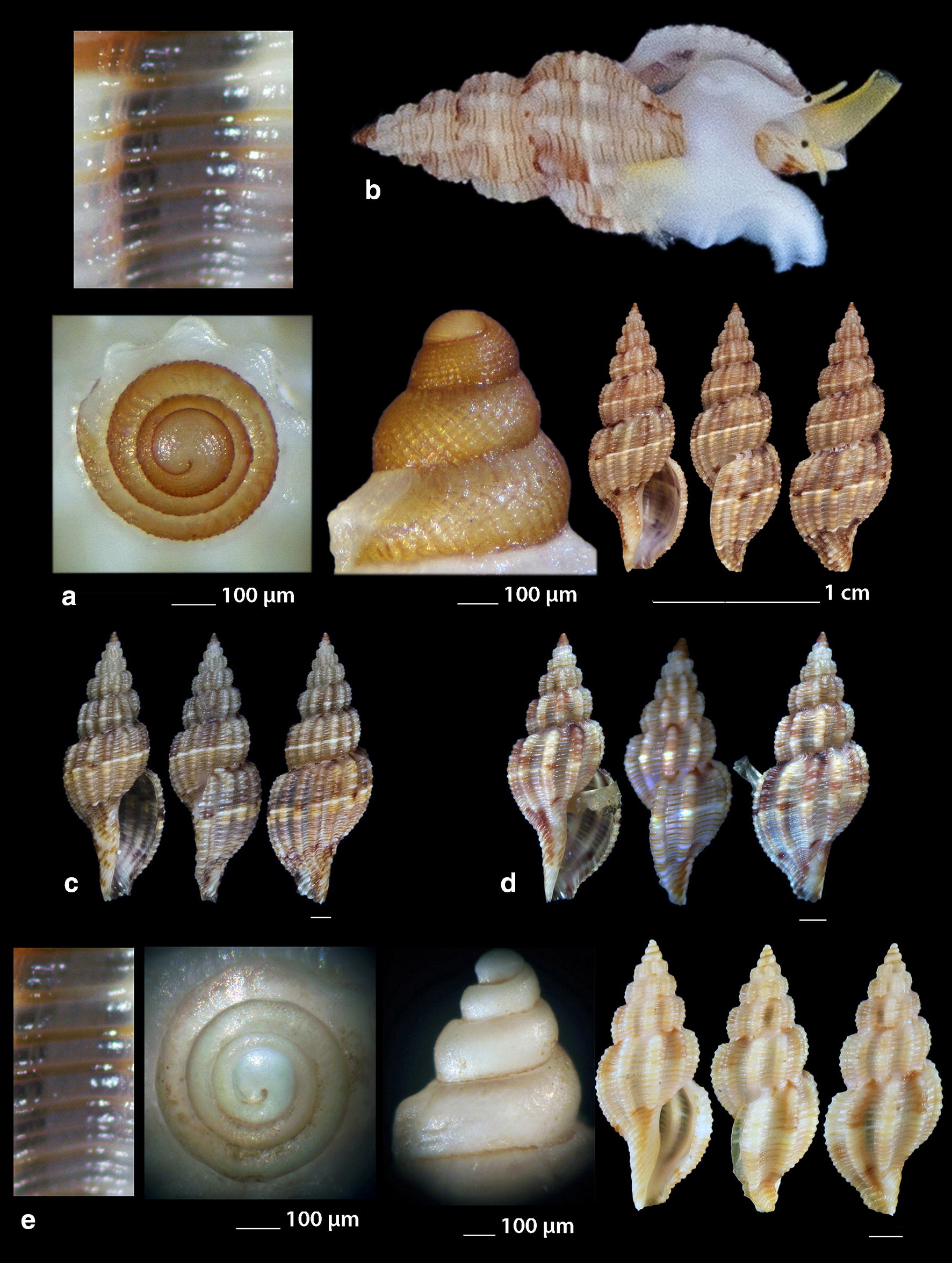
**a**–**e** *Raphitoma villaria*, **b** live specimen, **e** specimen with unusual protoconch. Bar = 1 mm, unless otherwise indicated

***Raphitoma villaria*** Pusateri & Giannuzzi-Savelli, 2008 (Fig. 18a–e)

*Collection stations* Sixteen live specimens and 28 shells (17.30–10.40 mm long, 6.45–4.25 mm wide), − 40/250 m, mixed bottoms, Central Thermaikos Gulf, 40°05′N–22°56′Ε; Central Singitikos Gulf, 40°11′N–24°02′Ε; Pyrgadikia, Chalkidiki, 40°18′N–23°45′Ε; Central Saronikos Gulf, 37°45′N–23°48′Ε; Korinthiakos Gulf, 38°19′N–22°19′Ε; Lavrio, Attiki, 37°41′N–24°06′E; Lefkos, Karpathos Island, 35°37′N–27°04′Ε.

*Description* Shell hyaline, fusiform, slender, thin and slightly more than 2.6 times as long as wide. Protoconch multispiral, mean 565 μm wide and 675 μm high, of mean 3.45 dome-shaped whorls the first of which with a sculpture of 8–9 spiral threads and covered with minute tubercles eventually giving rise to a clathrate sculpture which on its first 1/3 under the suture consists of only axial threads and on its lower 2/3 of diagonally crossing threads as well. Before the onset of the teleoconch, the last protoconch whorl exhibits of a weak keel. The teleoconch in mature individuals consists of 6.75 convex whorls separated by a deep suture and a narrow subsutural ramp exhibiting anal sinus marks. The body whorl occupies some more than 60% of the total length and bears 16–18 axial ribs, narrower than the interspaces, extending from the suture down to the shell base. Spiral sculpture of 26–27 narrow uninterrupted spiral cords, 12–13 situated above the aperture and the rest below it with a tendency to become stronger towards the tail of the shell. The inner wall of the shell viewed through the aperture exhibits a transparency. The suboval aperture occupies approximately 45% of the shell length, has a simple and internally smooth outer lip and a deep anal sinus. Background color cream-yellow to light lilac-yellow with a lighter narrow band on the lower third of the spire. Light honey-brown protoconch. Αnimal of white with light yellow highlights on the foot, the tentacles and the siphon.

Among the specimens examined, one (Fig. 18e) exhibits all the typical characteristics of the species but the protoconch which is smoother in its cancelated decoration and has the protoconch I prominent and dissociated from protoconch II, perhaps an abnormal development.

*Similar species R. villaria* bears a larger protoconch than *R. leufroyi* (600 μm vs 450 μm) of generally yellowish color (brown in *R. villaria*). The subsutural ramp is absent in *R. leufroyi*, the h/d ratio is higher in *R. villaria* (h/d > 2.5 vs < 2.2 in *R. leufroyi*). The spiral cordlets (16 in *R. villaria* vs 12 in *R. leufroyi*) are all of equal size in *R. villaria* vs of alternate size in *R. leufroyi*. The outer lip is thickened in *R. leufroyi* vs simple in *R. villaria*. The background color is lighter with brownish spots in *R. leufroyi* vs uniformly yellowish in *R. villaria*. The animal of *R. leufroyi* is pure white, with light blue blurs on the end of the foot. The eyes are larger in *R. leufroyi* and are placed halfway up the tentacles. The foot is wider in *R. leufroyi* while a radula is present in *R. leufroyi* but absent in *R. villaria* [47]. *Raphitoma villaria* is similar to *R. erronea* from which it differs in having a thinner shell, fewer spiral cords, a longer siphonal canal, a less rounded aperture, and an h/d ratio of nearly 3. *Raphitoma* *villaria* is also similar to *R. concinna* but the late is smaller, with fewer axial ribs and fewer but stronger spiral cords, typical brown cordlets on the spiral cords and light violet protoconch [47].

*Habitat and distribution* Known from the type locality (Taormina, East Sicily) and from Malta (Central Mediterranean Sea) [47].

*Status* Uncommon [47]. First documented record for the Hellenic Seas and the East Mediterranean Sea.

**Fig. 19:**
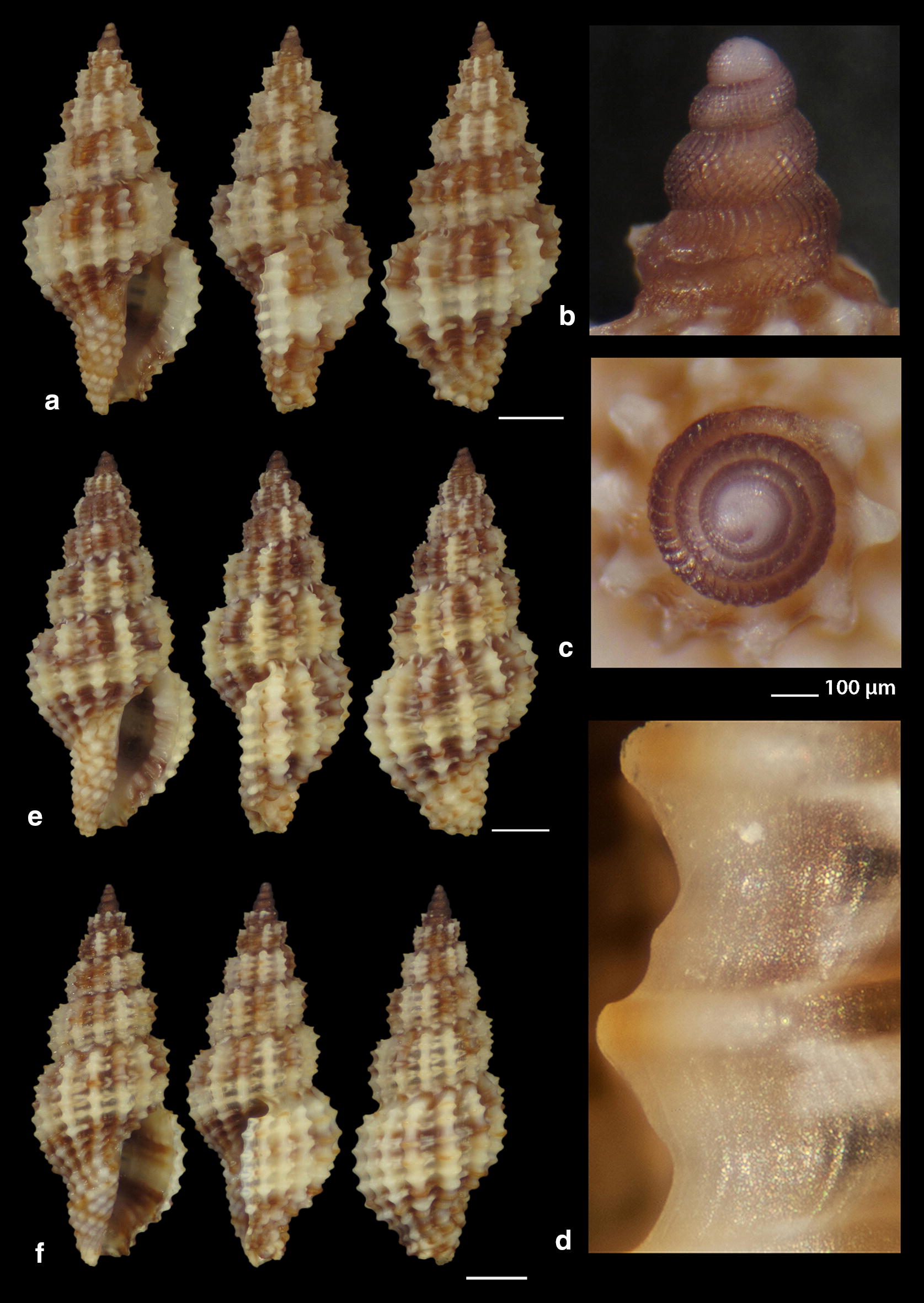
**a**–**d** *Raphitoma melitis* sp. n., **a** holotype (6.15 mm), **b**, **c** protoconch, **d** surface, **e** Paratype 1 (6.90 mm), **f** Paratype 2 (6.25 mm). Bar = 1 mm, unless otherwise indicated

**Fig. 20:**
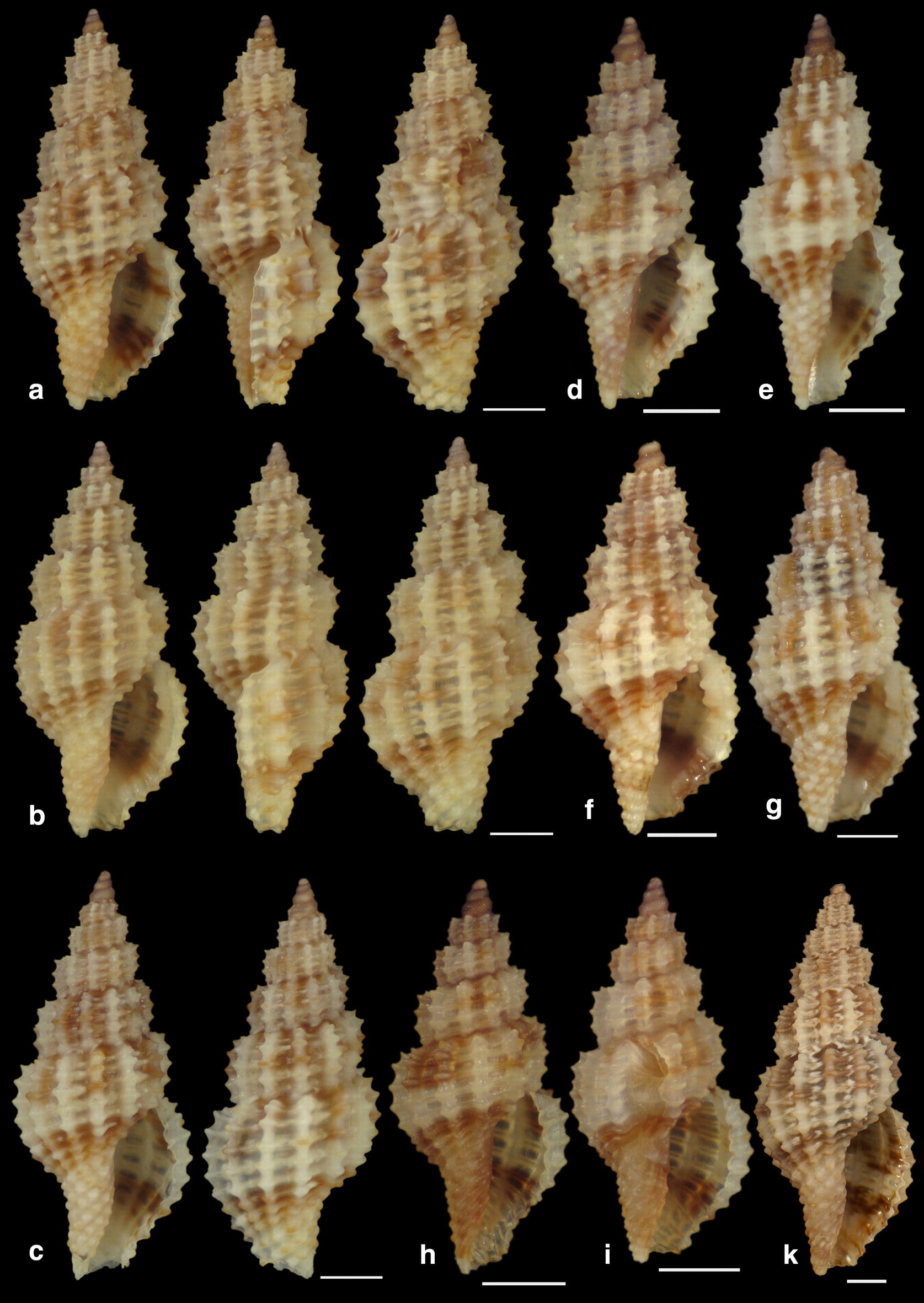
**a**–**j** *Raphitoma melitis* sp. n., **a** Paratype 3 (6.50 mm), **b** Paratype 4 (6.20 mm), **c** Paratype 5 (6.25 mm), **d**–**j** Paratypes 6–12, respectively. Scale Bar = 1 mm

**Fig. 21:**
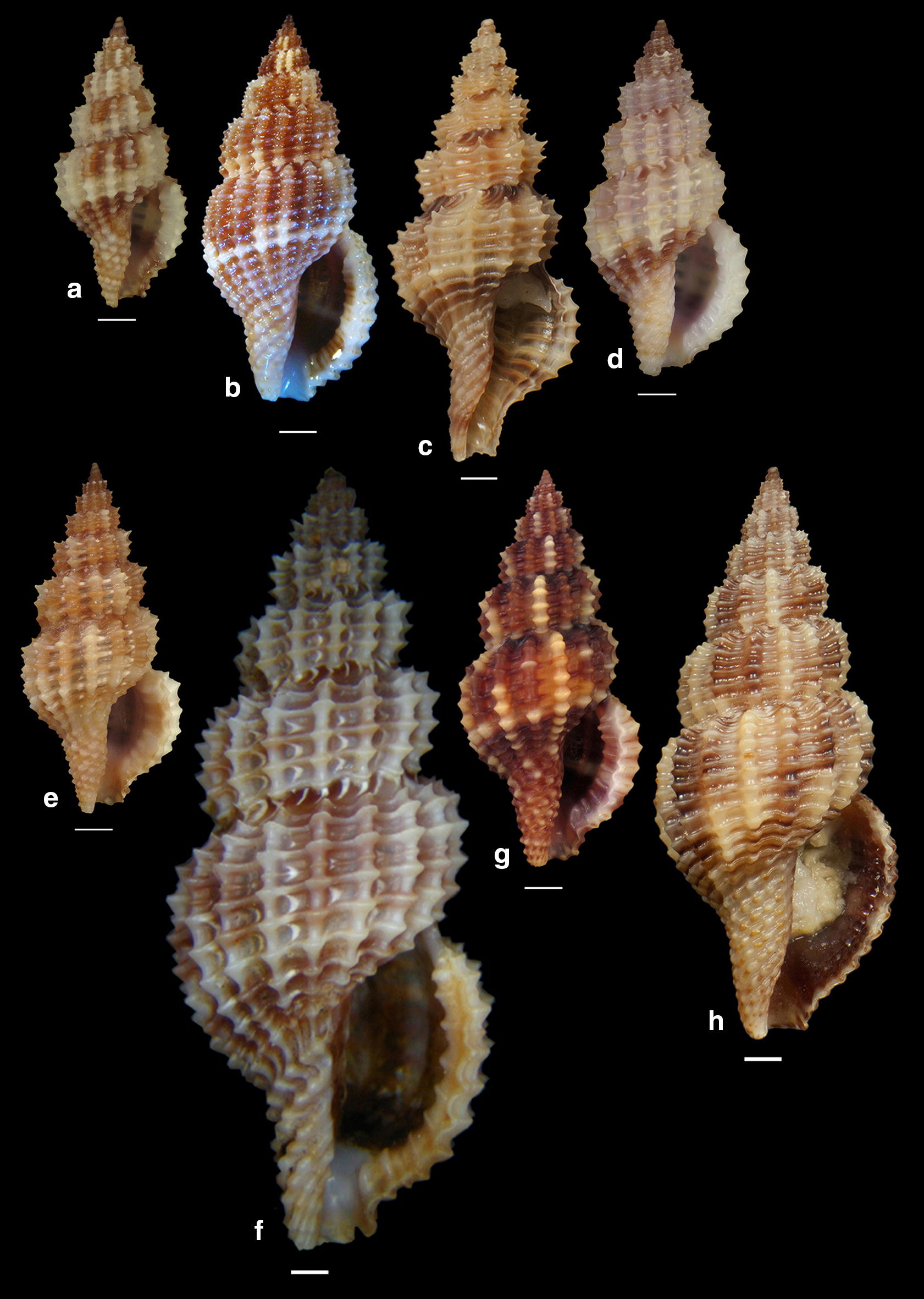
**a** *Raphitoma melitis* sp. n., **b** *Raphitoma bicolor*, C. *cordieri* (sensu Cossignani & Ardovini, 2011), **d** *Raphitoma echinata* (sensu *auctores*) Morphotype I, **e** *Raphitoma echinata* (sensu *auctores*) Morphotype II, **f** *Raphitoma echinata* (sensu auctores) Morphotype III, **g** *Raphitoma ephesina*, **h** *Raphitoma sophiae* sp. n. Scale bar = 1 mm

***Raphitoma melitis*** Kontadakis & Mbazios n. sp. (Figs. 19a–f, 20a–j, 21a)

*Description* Shell of small size for the genus. Holotype 6.15 mm × 2.85 mm, turreted, with high spire, porcelaneously opaque and nearly 2.1 times as long as wide. Multispiral protoconch, light purple-brown in color with whitish nucleus, 435 μm wide and 580 μm high, of 3.25 regularly cancellated and convex whorls, exhibiting on the last half whorl a weak keel. Embryonic shell (protoconch I) of 0.7 whorls, 160 μm in diameter, with sculpture of 5–6 spiral threads and covered with minute tubercles orderly placed on the threads. Protoconch II with fine clathrate sculpture which on the upper 1/3 of the whorl under the suture consists of only axial threads and on its lower 2/3 of diagonally crossing threads as well. The teleoconch of 5 convex whorls with a moderately wide ramp below the deep suture. The body whorl occupies ~60% of the total length, is inverted dome shaped, and bears 14 orthocline and narrow axial ribs with interspaces as wide as the ribs and 15 spiral cords much thinner than the ribs, 5 of which are situated on the penultimate whorl and the rest 11 down to the shell distinct tail. The spiral cords in their intersections with the axial ribs form acute, elongated and rectangular tubercles. The relatively high and widely spaced tubercles endow the shell with a spiky appearance. The aperture is elongated and oval, occupies 40% of the shells length and exhibits a rough and sinuous columella in its lower part, angled at its upper part. The anterior siphonal canal is long and wide while the posterior one is deep and narrow. The outer lip exhibits externally the endings of the 11 spiral cords, while, internally, 8 conspicuous and a equally spaced denticles. Shell surface granular and dull, with evident but smooth incremental scars. Background color cream white with irregularly placed light brown areas and with cream white some irregularly situated groups of/or isolated tubercles. The body whorl bears a cream-white suprasutural double spiral band and a cream white tail.

*Type material* Holotype (Fig. 19a–d), 6.10 × 2.85 mm, Central Saronikos Gulf (Greece), − 100 m, 37°44′N–23°48′Ε (CCK); Paratype 1 (Fig. 19e), 6.88 × 3.05 mm, West Saronikos Gulf (Greece), − 100 m, 37°52′N–23°17′E (CGMb); Paratype 2 (Fig. 19f), 6.25 × 2.55 mm, Central Saronikos Gulf (Greece), − 70 m, 37°54′N–23°39′E (CGZ); Paratype 3 (Fig. 20a), 6.50 × 2.75 mm, Thermaikos Gulf (Greece), − 70 m, 39°57′N–23°02′E (CGP); Paratype 4 (Fig. 20b), 6.20 × 2.75 mm, Thermaikos Gulf (Greece), mixed bottom, − 5 m, 40°23′N–22°53′Ε (CTM); Paratype 5 (Fig. 20c), 6.25 × 2.80 mm, Thermaikos Gulf (Greece), mixed bottom, − 10 m, 40°23′N–22°53′Ε (CTM); Paratype 6 (Fig. 20d), 5.20 × 2.30 mm, Saronikos Gulf (Greece), − 70 m, 37°54′N–23°39′E (CGZ); Paratype 7 (Fig. 20e), 5.30 × 2.80 mm, Saronikos Gulf (Greece), − 60 m, 37°45′N–23°48′Ε (CCK); Paratype 8 (Fig. 20f), 5.80 × 2.75 mm, West Saronikos Gulf (Greece), − 100 m, 37°52′N–23°17′E (CGMb); Paratype 9 (Fig. 20g), 6.65–2.50 mm, Saronikos Gulf (Greece), − 70 m, 37°45′N–23°48′Ε (CCK); Paratype 10 (Fig. 20h), 4.85 × 2.25 mm, South Saronikos Gulf (Greece), − 100 m, 37°40′N–23°14′Ε (CGMb); Paratype 11 (Fig. 20i), 4.90 × 2.35 mm, South Saronikos Gulf (Greece), − 100 m, 37°40′N–23°14′Ε (CGMb); Paratype 12 (Fig. 20j), 10.35 × 4.30 mm, Central Thermaikos Gulf (Greece), − 40 m, 40°03′N–23°07′E (CGP).

*Type localities* Aegean Sea (Saronikos and Thermaikos Gulfs), − 5/100 m, on rocky-biogenic bottoms.

*Distribution* Known only from the type localities.

*Habitat* The specimens were collected from bottom material on small scale fishing nets cast on rocky-biogenic substrates in two different and distant, by nearly 500 km, localities.

*Etymology* This species is named after the newly born daughter Melite (a Nereid) of our team’s colleague DVM Constantinos Kontadakis.

Remarks

*Raphitoma melitis* n. sp. (Fig. 21a) can be compared with: *R. bicolor* (Risso, 1826) (Fig. 21b); *R. cordieri* (sensu Cossignani & Ardovini, 2011) (Fig. 21c); *R. echinata* (sensu *auctores*) Morphotype I (Fig. 21d); *R. echinata* (sensu *auctores*) Morphotype II (Fig. 21e); *R. echinata* (sensu *auctores*) Morphotype III (Fig. 21f); *R. ephesina* Pusateri, Giannuzzi Savelli & Stahlschmidt, 2017 (Fig. 21g); *R. sophiae* Kontadakis & Polyzoulis n. sp. (Fig. 21h). See also Table 1 for a more detailed comparison. More specifically, it differs from *R. bicolor* as *R. melitis* sp. n. is slender, has fewer and more narrow axial ribs and thinner spiral cords, fewer cords over the suture, fewer aperture denticles, a longer tail and is smaller by almost half. It is different from *R. cordieri* (sensu Cossignani & Ardovini, 2011) and all *R. echinata* forms as it is less spiky and, in particular, from: *R. cordieri* (sensu Cossignani & Ardovini, 2011) in its fewer spiral cords and aperture denticles, the less wide subsutural ramp and the smaller—by half-size; *R. echinata* f. I in its longer tail, its moderately wide subsutural ramp and the smaller—by half-size; *R. echinata* f. II in its longer tail, its subsutural ramp that bears radial color brush- strokes and its more turreted; *R. echinata* f. III in its fewer axial ribs and spiral cords, its long tail, the subsutural ramp, the different coloration and its smaller by 1/3 size. It also differs from *R. ephesina* in its slender shell, the fewer spiral cords on the penultimate whorl, its longer tail, the wider subsutural ramp, the subsutural ramp radial whitish brush-strokes and the coloration. Finally, *R. sophiae* sp. n. bears more spiral cords than *R. melitis* sp. n., a shorter tail, a more narrow subsutural ramp, is much longer and lacks the spiky appearance of *R. melitis* sp. n. In conclusion, *R. melitis* sp. n. inhabits the same environment as all other Mediterranean *Raphitoma* species and can easily be distinguished from its sympatric congenerates (Fig. 21a–h).

**Table 1:** Comparison of *R. melitis* n. sp. characteristcs of mature specimens against those of *R. bicolor*, *R. cordieri* (sensu Cossignani & Ardovini), *R. echinata* f. I, *R. echinata* f. II, *R. echinata* f. III, *R. ephesina* and *R. sophiae* n. sp.

Species/characteristics	*R. melitis* sp. n.	*R. bicolor*	*R. cordieri*	*R. echinata* f. I	*R. echinata* f. II	*R. echinata* f. III	*R. ephesina*	*R. sophiae* sp. n.
Length/width ratio	2.1–2.4	2.4–2.7	2.4	2.1–2.5	2.2–2.4	2.2–2.4	2.2–2.3	2.4–2.5
Number of axial ribs on body whorl	12–14	16–18	11–12	13–14	12–13	15–16	10–11	18
Axial ribs form	Convex	Convex	Convex	Very convex	Slightly convex	Very convex	Convex	Very convex
Number of spiral cords on body whorl	14–16	15–18	17–18	13–14	15	16–17	17–18	25
Number of spiral cords on the penultimate whorl	5–6	9–7	4–5	4–5	4–5	6	5–6	10
Aperture teeth	7–8	9–10	10–12	8–11	7–8	9	7–8	12
Tail	Moderately long	Short	Long	Short	Moderately long	Moderately long	Short	Moderately long
Teleoconch microsculpture	Sandpaper appearance	Smooth	Smooth	Smooth	Sandpaper appearance	Sandpaper appearance	Smooth	Smooth
Subsutural ramp	Moderately wide	Narrow	Very wide	Wide	Very wide	Very wide	Very narrow	Moderately wide
Body whorl curvature	Inverted dome shaped	Rounded	Rounded	Rounded	Inverted dome shaped	Inverted dome shaped	Rounded	Inverted dome shaped
Teleoconch color pattern	Randomly placed light brown patches on light beige background and 2 light beige cords over the suture of the last whorls	Randomly placed cream-white patches on honey-yellow to deep purple background and white rib pairs	Uniformly beige with a brown band as a prolongation of the suture	Randomly placed diffused light brown, light purple and white areas on a light beige background	Randomly placed light brown patches and white strokes on light beige background	Randomly placed light brown patches and white strokes on light beige background	Background color cherry-red except an over sutural cordlet and randomly placed areas which are cream-white	Randomly placed light brown patches on light beige background
Subsutural ramp radial color brush strokes	Present	Absent	Present	Present	Absent	Present	Absent	Present
Size (mm)	4.85–10.35	10.30–12.50	12.30–15.00	10.60–13.00	8.10–10.90	19.10–21.10	8.90–11.00	9.30–15.55

**Fig. 22:**
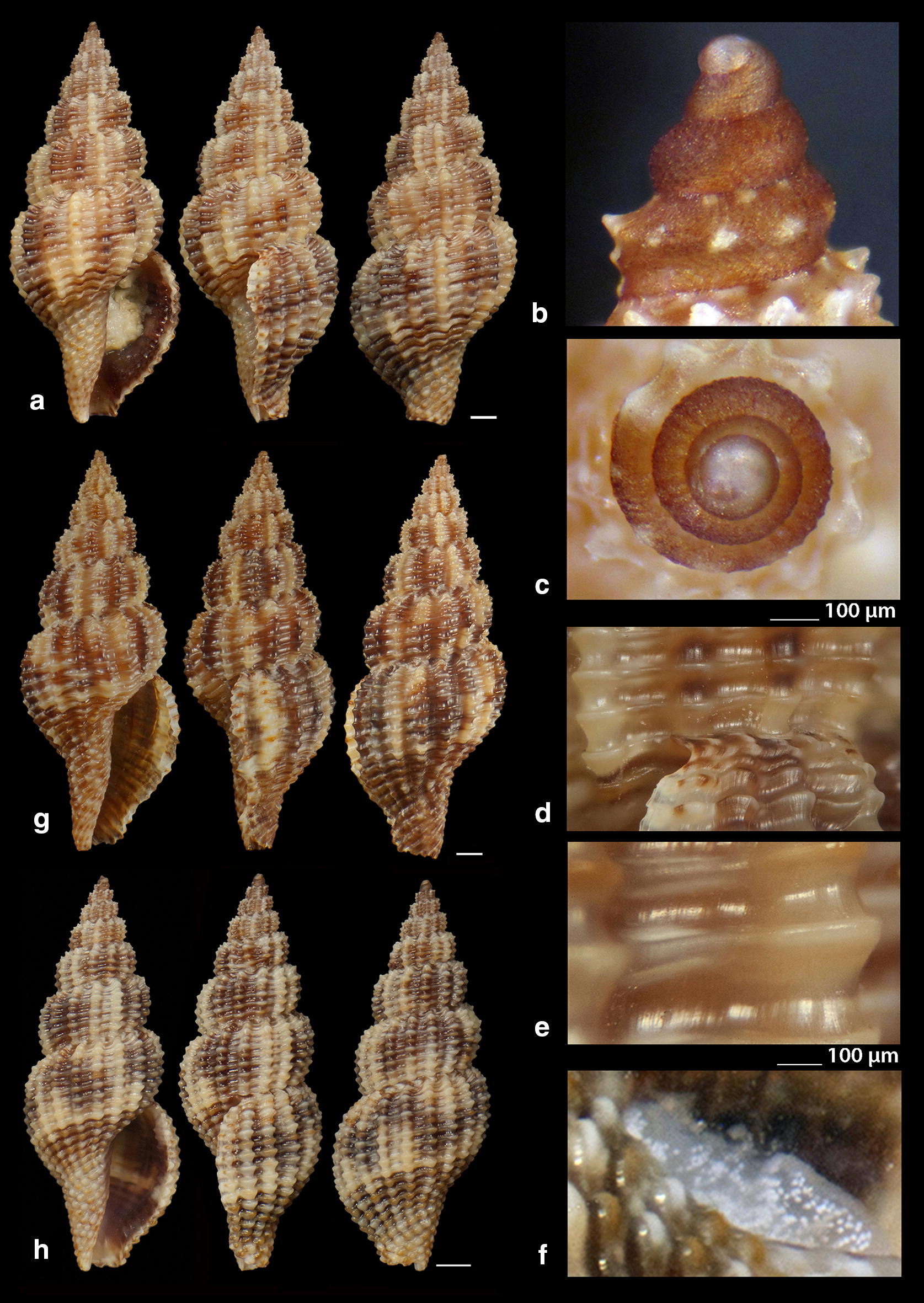
**a**–**f** *Raphitoma sophiae* sp. n., **a** holotype (15.55 mm), **b**, **c** protoconch, **d** ornamentation by the posterior siphonal canal, **e** surface, **f** animal foot, **g** Paratype 1 (15.10 mm), **h** Paratype 2 (11.25 mm). Scale bar = 1 mm

**Fig. 23:**
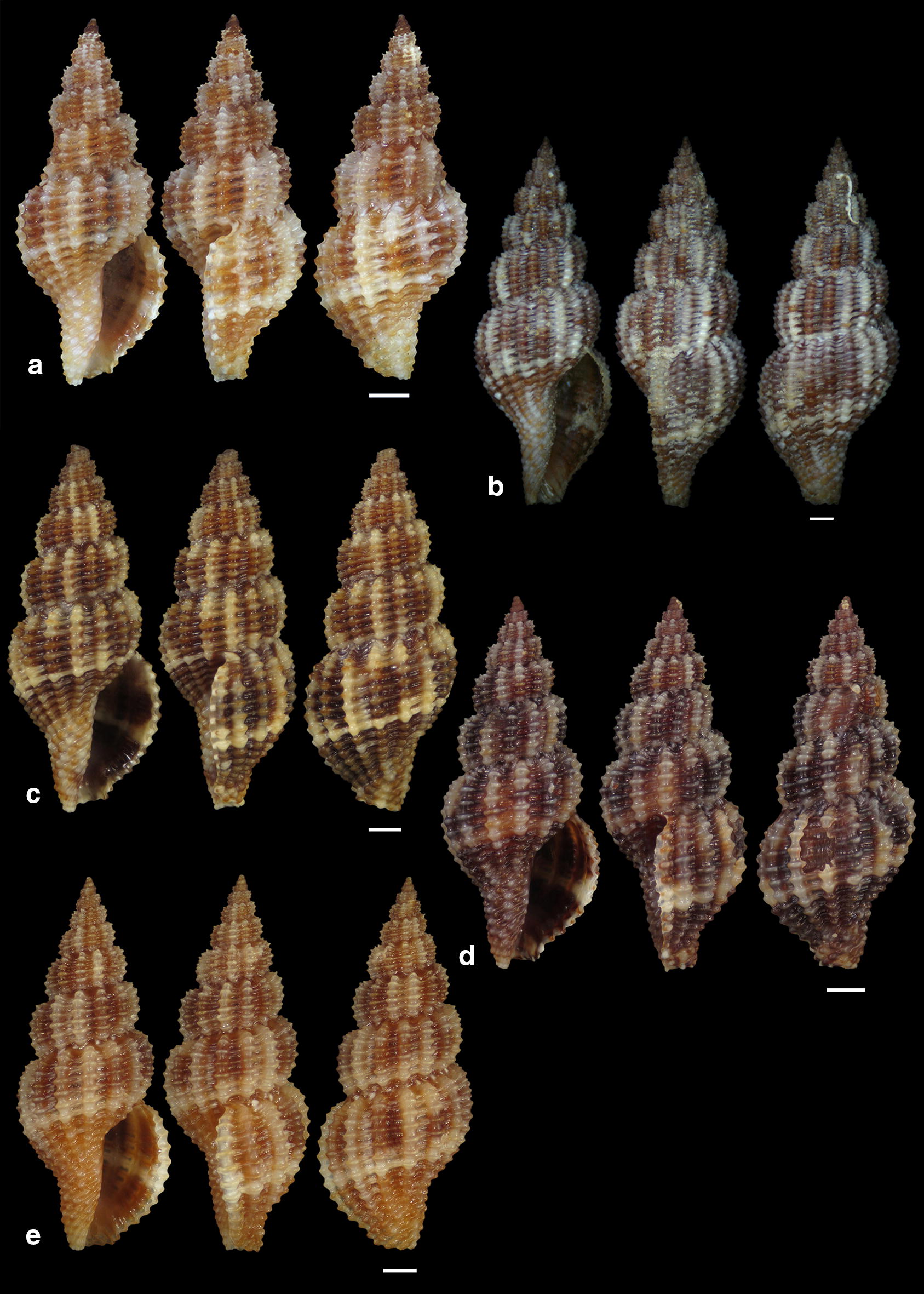
**a**–**e**. *Raphitoma sophiae* sp. n., **a** Paratype 3 (9.30 mm), **b** Paratype 4 (15.15 mm), **c** Paratype 5 (11.25), **d** Paratype 6 (9.75), **e** Paratype 7 (11.30). Scale bar = 1 mm

***Raphitoma sophiae*** Kontadakis & Polyzoulis n. sp. (Figs. 22a–h and 23a–e)

*Description* Shell of medium size for the genus. Holotype 15.55 mm × 6.56 mm, fusiform, porcelaneously opaque and almost 2.4 times as long as wide. Multispiral protoconch, light brown in color, 450 μm wide and 545 μm high, of 3 regularly cancellated and convex whorls, exhibiting on the last half whorl a weak keel with 4 white spots. Embryonic shell (protoconch I) of 1 whorl, with of lighter color nucleus and 200 μm of diameter. The teleoconch consists of 7.25 highly convex whorls separated by a conspicuous suture. Moderately wide subsutural ramp. The body whorl occupies 60% of the total length, is inverted dome shaped, and bears 18 orthocline and narrow axial ribs with interspaces as wide as the ribs and 25 spiral cords thinner than the ribs, 10 of which are situated on the penultimate whorl and the rest 15 down to the distinct tail of the shell. The spiral cords in their intersections with the axial ribs form smooth, elongated and rectangular tubercles. The relatively high number of ribs and spirals in combination with the tubercles endow the shell with a very dense, though smooth appearance. The first three adapical cords increase gradually in size, are close to each other and form an oblique shoulder immediately bellow the suture. The aperture is elongated oval, occupies 43% of the shell length and exhibits a smooth and slightly sinuous columella in its lower part, angled at its upper part. The anterior siphonal canal is long and wide while the posterior one is deep and narrow. The outer lip exhibits externally the endings of the 18 spiral cords, while, internally, 12 conspicuous and regularly positioned teeth. Shell surface smooth and bright, almost glossy, with evident but smooth incremental scars. Background color light beige with irregularly placed brown areas and with lighter the tubercles and some irregularly situated groups of/or isolated orange tubercles. The body whorl bears at its middle a lighter color spiral band as a prolongation of the suture and the shoulder below the suture exhibits alternating lighter and darker in color radially placed brush strokes. The animals exhibited an of white foot with dense white dots and a uniformly grey-black siphon.

*Type material* Holotype (Fig. 22a–f), 15.55 × 6.56 mm, Central Thermaikos Gulf (Greece), Potidea, rocky-biogenic bottom, − 50/60 m, 40°09′N–23°08′E (CGP); Paratype 1 (Fig. 22g), 15.10 × 6.13 mm, Central Thermaikos Gulf, Potidea (Greece), rocky-biogenic bottom, − 50/60 m, 40°09′N–23°08′E (CGP); Paratype 2 (Fig. 22h), 11.80 × 4.65 mm, Central Thermaikos Gulf, Potidea (Greece), rocky-biogenic bottom, − 50/60 m, 40°09′N–23°08′E (CGP); Paratype 3 (Fig. 23a), 9.30 × 3.80 mm, Central Saronikos Gulf (Greece), rocky-coralligenous bottom, − 50/70 m, 37°45′N–23°48′Ε (CCK); Paratype 4 (Fig. 23b), 15.15 × 5.25 mm, Siggitikos Gulf (Greece), rocky-biogenic bottom, − 60/70 m, 40°09′N–22°48′Ε (CDM); Paratype 5 (Fig. 23c), 11.25 × 4.80 mm, Souda, Crete (Greece), rocky-biogenic bottom, − 40/60 m, 35°28′N–24°06′Ε (CGZ); Paratype 6 (Fig. 23d), 9.75 × 4.20 mm, Central Saronikos Gulf (Greece), rocky-biogenic bottom, − 50 m, 37°54′N–23°39′E (CPO); Paratype 7 (Fig. 23e), 11.30 × 4.65 mm, Central Saronikos Gulf (Greece), rocky-biogenic bottom, − 60 m, 37°41′N–23°35′E (CPO);

*Type localities* Aegean Sea (Saronikos and Thermaikos Gulfs) and Sea of Crete, − 40/70 m depth on rocky-biogenic bottom.

*Distribution* Known only from the type localities.

*Habitat* The specimens were collected live from bottom material on small scale fishing nets cast on rocky-biogenic substrates in three different and distant, up to 1000 km, localities.

*Etymology* This species is named after our team’s lady colleague Prof. Sofia (Sophia) Galinou-Mitsoudi.

Remarks

**Fig. 24:**
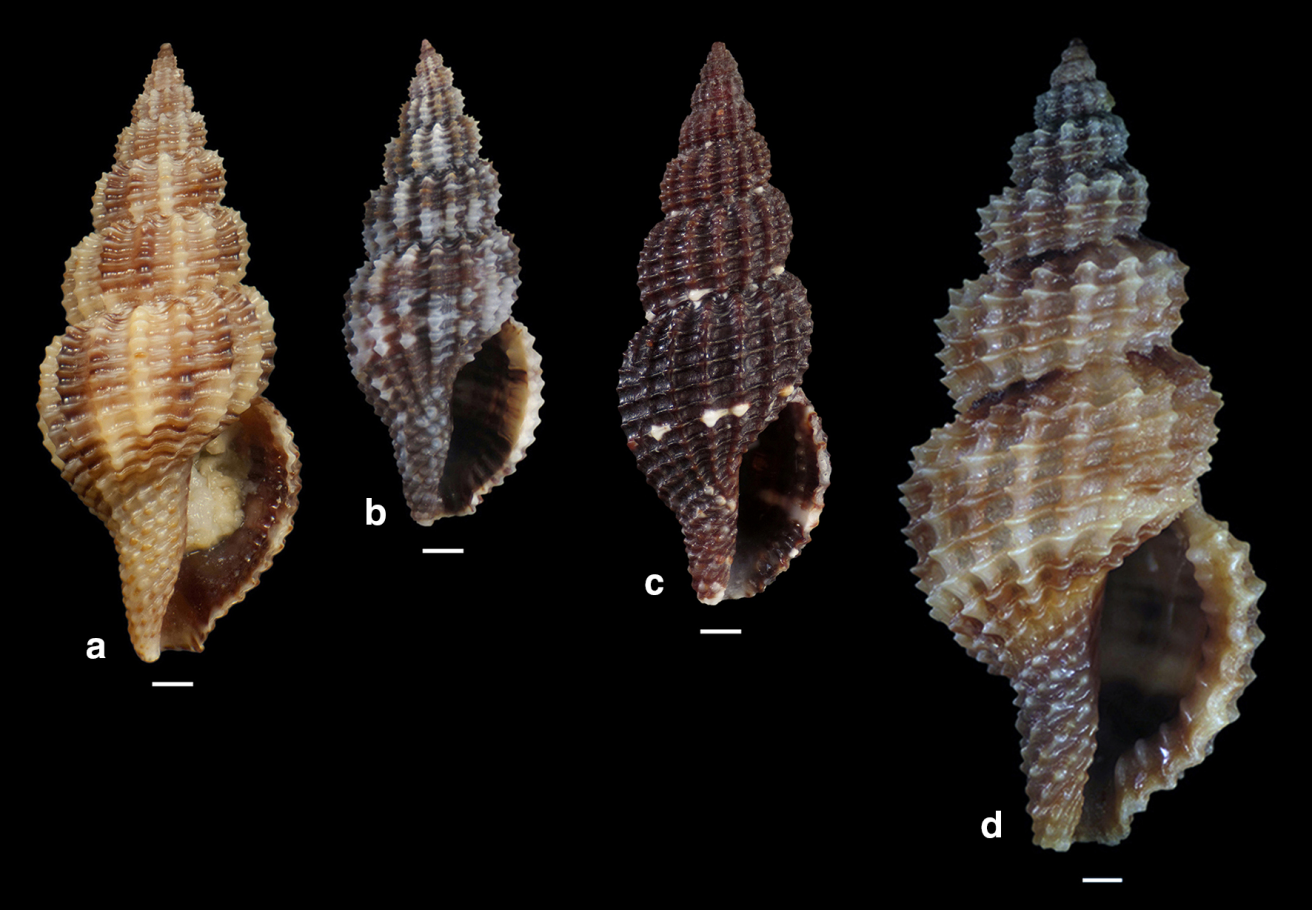
**a** *Raphitoma sophiae* sp. n. holotype (15.55 mm), **b** *Raphitoma densa* (11.65 mm), **c** *Raphitoma atropurpurea* (13.50 mm), **d** *Raphitoma echinata* (sensu *auctores*) Morphotype IV (20.95 mm). Bar = 1 mm

*Raphitoma sophiae* n. sp. can be compared with *R. densa*, *R*. *atropurpurea* and *R. echinata* (sensu *auctores*) Morphotype III (Fig. 24a–d). See also, Table 2, for a more detailed comparison. More specifically, it differs from: *R. densa* (Fig. 24b) in its bigger size, the l/w ratio (*R*. *densa* 2.8 from Pusateri et al. [16], its more convex whorls, the higher number of spiral cords (25) on the body whorl, the wider spaces between the axial ribs on respective whorls, the more evident subsutural ramp, the longer tail, the absence of microgranules on the microsculpture of the teleoconch, and in its very characteristic color pattern; *R. atropurpurea* (Fig. 24c) is more slender [16, 45], has less convex whorls, less spiral cords on the body whorl, shorter tail and always a uniform background color; *R. echinata* (sensu auctores) Morphotype III (Fig. 24d) exhibits a much more spiny appearance, a wider subsutural ramp, a smaller number of spiral cords and axial ribs on the body whorl, and a different color pattern. The substrate of the new species is typical for the members of the genus as they feed upon polychaete worms that contribute to its formation [22]. In conclusion, *Raphitoma sophiae* sp. n. inhabits the same environment as all other Mediterranean *Raphitoma* species and can easily be distinguished from its sympatric congenerates.

**Table 2:** Comparison of *Raphitoma sophiae* n. sp. characteristcs of mature specimens against those of *R. densa*, *R. atropurpurea* and *R. echinata* f. III

Species/characteristics	*R. sophiae*	*R. densa*	*R. atropurpurea*	*R. echinata* f. III
Length/width ratio	2.4–2.5	2.5–2.8	2.6–2.8	2.2–2.4
Number of axial ribs on body whorl	18	18	17–23	15–16
Axial ribs form	Very convex	Convex	Convex	Very convex
Number of spiral cords on body whorl	25	19–21	19–21	16–17
Number of spiral cords on the penultimate whorl	10	7–9	8–9	6
Aperture teeth	12	11	11–12	9
Tail	Moderately long	Short	Short	Moderately long
Teleoconch microsculpture	Smooth	Rough (microgranules)	Smooth	Sandpaper appearance
Subsutural ramp	Wide	Moderately wide	Narrow	Very wide
Body whorl curvature	Inverted dome shaped	Rounded	Rounded	Inverted dome shaped
Teleoconch color-pattern	Randomly placed light brown patches on light beige background	Dense patches of dark grey or cerulean on a dull background	Uniformly dark brown-purple with white cord as an extension of the suture	Randomly placed diffused light brown areas on light beige background
Subsutural ramp radial color brush strokes	Present	Absent	Absent	Present
Size (mm)	9.30–15.55	9.60–11.80	8.00–17.00	19.10–21.10

Genus: ***Taranis*** Jeffreys, 1870

Type species *Trophon moerchii* Malm, 1861 by original designation

**Fig. 25:**
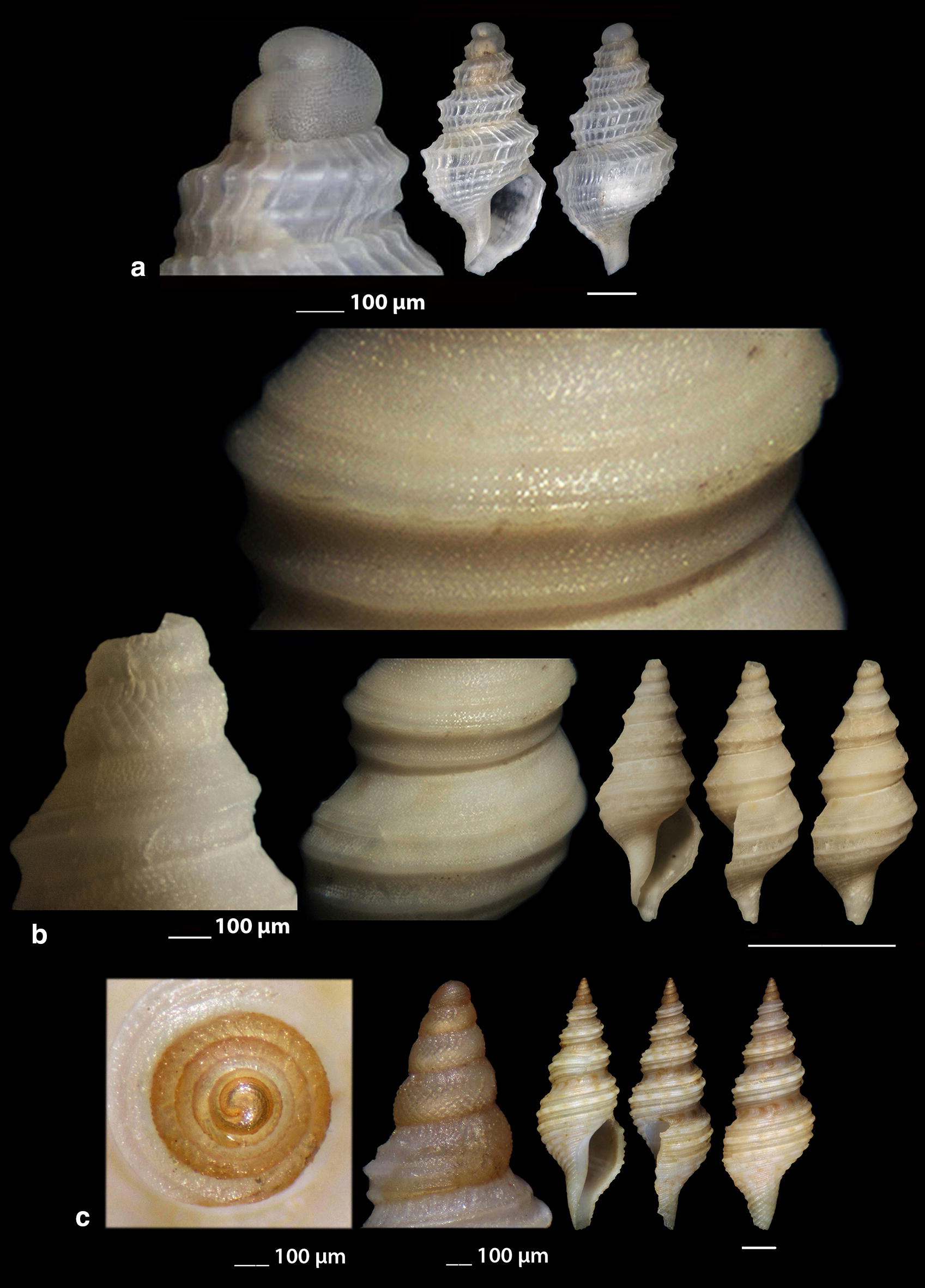
**a** *Taranis moerchii*, **b** *Teretia* cf. *neocaledonica*, **c** *Teretia teres*. Bar = 1 mm, unless otherwise indicated

***Taranis moerchii*** (Malm, 1861) (Fig. 25a)

Genus: ***Teretia*** Norman, 1888

***Teretia*** cf. ***neocaledonica*** Morassi & Bonfitto, 2015 (Fig. 25b)

*Collection station* One shell (1.75 mm long, 0.70 mm wide), white coral bed, − 400 m, Lemnos Island, 40°05′N–25°12′E.

*Description* The minute fusiform shell exhibits a high spire with excavated base and long anterior canal. Its partially broken protoconch is conical of 2 remaining whorls with diagonally cancellate sculpture terminating shortly above the abapical linear suture. Subsutural ramp wide, slightly concave. First 2 teleoconch whorls with a prominent peripheral keel below the mid-height of the whorl. Body whorl with 3 main cords and additional weak cords: one on abapical part of subsutural ramp and 1–2 in each interspace between main spiral cords. Its base and rostrum bear about 20 spiral cords. Teleoconch whorls exhibit fine axial growth lines with granules. Aperture lanceolate with thin outer lip. Shell yellowish-white with few weak, light brown, subsutural blotches on the last whorl.

*Similar species T.* cf. *neocaledonica* resembles the fossil *T. turritelloides* (Bellardi, 1847) in its shape and position of the spiral cords but differs from the latter in the sculpture of the protoconch and the teleoconch. In particular, in *T.* cf. *neocaledonica* the axial riblets of each protoconch whorl strongly cancelate on the periphery, while in *T. turritelloides* cancelation occurs on the whorl surface except for a narrow area near adapical suture. In addition, *T. turritelloides* bears a more conspicuous secondary sculpture than *T.* cf. *neocaledonica*. *Teretia* cf. *neocaledonica* also differs from the fossil *T. elegantissima* (Foresti, 1868) in having a much less prominent peripheral keel [48]. Compared with *T. fusianceps* Nordsieck, 1972, *T.* cf. *neocaledonica* bears 3 carinas instead of 2, body whorl with 3 main cords and additional weak cords instead of 5 and *T.* cf. *neocaledonica* bears surface granules that are absent in *T. fusianceps* [39, 48].

*Habitat and distribution* Insufficient data for the Indo-Pacific Ocean as it is described only from New Caledonia.

*Status* Insufficient data due to its rarity. First record for the Mediterranean Sea.

***Teretia teres*** (Reeve, 1844) (Fig. 25c)

## Discussion

This work raises the Hellenic gastropod biodiversity from 654 species [28, 32, 49–51] to 664 species, while the Raphitomidae family is enriched by 45%.

The taxonomy of shelled mollusks was, and continues to be, almost exclusively based on shell morphology. For almost 150 years of scientific research on the family, shell characters have proved to be rather effective species-level means of identification, especially when the protoconch is intact. Furthermore, reliance on shell characters is particularly justified by the fact that many species have never been encountered alive—a fact that especially holds true for Raphitomidae. As a result, the majority of the described species of the family are represented by shells, thus leaving shell morphological characters as the major source of evidence for their taxonomy. Dealing with the Raphitomidae family and the genus *Raphitoma*, in particular, the need for a practical identification tool emerges, as the application of the “morphological species” concept, apparently, exhibit certain difficulties, without mentioning the labyrinth of synonyms and misidentifications. The more comprehensive relevant monographs up to recently seemed to be that of Nordsieck [39] and that of Cachia et al. [21], but unfortunately the presentation in the form of sketches diminishes the validity of those publications. Thus, the ongoing revision of the Mediterranean members of the genus *Raphitoma* by Pusateri et al. [16–20] and the presentation of certain species by Gofas et al. [22] are considered the best guides to the Mediterranean members of the group. Apart from research gaps, there is a lack of useful tools for identification, especially considering the Greek fauna, such as publications with full descriptions and comparisons of the species, and an extreme shortage of detailed and high quality images.

The decisive stage of any taxonomic outcome in malacology is the critical evaluation of the shell characters with the aim to designate and exemplify reliable discriminating features. Towards this direction, in the last few years, protoconch characteristics have been almost exclusively used for the systematics of some Mediterranean, so called, “sibling” *Raphitoma* species [16–20]. When, though, someone examines the characteristics of either the paucispiral-lecithotrophic or the multispiral-planktotrophic protoconches of these members of the genus, is prompted to the conclusion that there are not fundamental differences between the protoconches of the different species within the same group of either planktotrophic or lecithotrophic development. This lack of significant differences together with the limited interspecific variability related either to the number of the whorls or the microsculpture of the protoconch, make rather impossible the definite characterization of a species exclusively on the bases of its protoconch’s characteristics.

Color pattern is often the first character used in identifying a shell, but even with living specimens this might be misleading because of extensive variability. In long dead shells or those stored in ethanol, the colors rapidly fade. Apart from that, teleoconch or protoconch interspecific color polymorphism observed in certain species, diminishes the validity of the use of that character for taxonomy (Figs. 1, 4, 5, 7, 11, 12, 13, 14, 15, 16, 18, 22, 23). The same may also hold with the color of the animal itself as the only difference observed between species is that of the hue of the rear siphon or it may even very within members of the same species as in the case of *R. atropurpurea* (Fig. 4).

Protoconch type, teleoconch macro- and microsculpture and relative length of the siphonal canal, are also useful, but rather variable, characters for distinguishing between species. However, all of these are hard to describe in a way that is helpful for species identification. The micro- and macro-sculpture may be the most useful of these, but sometimes even these are hard to interpret. The transition zone between protoconch and teleoconch is occasionally a useful morphological detail to confirm the determination of a doubtful specimen [42]. This is especially useful, for instance, to distinguish between *R. aequalis* and *R. linearis*. The later has the upper two teleoconch whorls of a dark purplish-brown color before those of a much lighter hue. The sibling species of the group are in need of DNA-based phylogeny. When DNA-based analyses are produced, it might also very well turn out that some Greek species are different from their Mediterranean congenerics. Available evidence indicates that most Mediterranean species of the genus *Raphitoma* are confined to the Mediterranean, but species of the *linearis* complex as well as the *R. leufroyi* group are exceptions being encountered from Norway to Canary Islands, as well as both south and north of the Mediterranean Sea [42].

Of the 28 species of *Raphitoma* shown and/or described, only 7 are relatively common in the Hellenic waters. This publication does not claim that has exhausted the diversity of the members of the genus in the area. The two new species described as well as several undescribed less well preserved or immature shells indicate that the list of Greek species is longer than those named above and points towards a larger species pool than suspected so far. Especially *R*. *echinata* complex will most likely be included in future reviews of the Greek raphitomid fauna as it may harbor a number of new species emerging out of DNA molecular analysis. In this publication, part of the reason for the hesitation to formally increase the number of species belonging to the local fauna is also the lack of live collected specimens that would greatly facilitate that analysis.

The collection and identification for the first time of live individuals of *R. atropurpurea* and *R. villaria* as well as of the many shells of other Raphitomid species indicates not only that Greek waters comprise a hospitable environment for species of the kind but also that focused search on biogenic and maerl material could lead to a further enrichment of the recorded biodiversity of the area.

The collection of a specimen of *Teretia* cf. *neocaledonica* from the North Aegean Sea came as a surprise and raised the question as to whether it represented a recent shell or a fossil. As it was found in a rather good condition for its size and on a white coral bed that is the expected background for members of the group [22], we are inclined to consider the finding as recent. The species was lately reported and described as new from the waters of New Caledonia [48], thus making the finding more improbable if its origin was attributed to lessepsian or ship ballast migration. The resemblance between the recent Indo-Pacific species described in Morassi & Bonfitto [48] and members of *Teretia* from the Tertiary of Europe suggests a Tethyan origin for the genus. Despite their extensive survey in the literature the authors were unable to trace *Teretia* species in the Tertiary of the Indo-Pacific region. That may be supportive to the hypothesis that the genus had a wide East–West distribution, i.e. from the eastern sectors to the western ones of the extended biogeographic unit defined as Tethys Realm, until the late Early Miocene, when the Atlantic and Mediterranean sector broke down from the Indo-Pacific one [52, 53]. Under this hypothesis, the possibility that an isolated subpopulation of the species survived the geological eras till now in some Mediterranean depths, may stand. Although we are rather confident of our identification we present it as *T*. cf. *neocaledonica* because to its non intact protoconch. We should also add the follow commentaries: a. Due to the small size and the depth distribution of the members of the genus *Teretia*, the species may have escaped the attention of the researchers, b. The smaller size of our specimen to that of *T*. *neocaledonica* may be attributed to unfavorable conditions in a new environment, c. The striking resemblance of our specimen to *T*. *neocaledonica* as that was accepted by Dr. Francesco Pusateri (personal communication) encourages us to be confident of our identification, d. The inclusion of our specimen in this publication would be essential for future comparisons with new findings, e. As a new species, the distribution of *T*. *neocaledonica* is unknown, f. Shells of this size are of no commercial value.

Considering the *R. echinata* complex, the application of molecular analysis is necessary, but the technique is conclusive only in case of positive results and needs alive and plentiful material. Reciprocal cooperation between research teams across the Mediterranean is, therefore, of paramount importance.

If biological material is available, molecular techniques are currently employed in taxonomic analysis and are revealing cases of cryptic species in all groups of animals, including mollusks (e.g. [54–58]). Again, molecular data are used in combination with shell and anatomical characters and occasionally become the final proof of the existence of separate species. This latter could unavoidably be proved true, as the molecular analysis is decisive only above a certain factor of genetic divergence. Thus, if two species are recently genetically isolated, the distinction between them could be impossible even with the traditional morphological approach. Nevertheless, the objective of our future work from now on is to collect sufficient live specimens for mtDNA markers analysis as well as interbreeding experiments that could bring an end to existing deadlocks.

## Conclusions

By this report, the Hellenic *Raphitomidae* biodiversity is enriched by 10 new records, out of which, two are new species, one is new record for the Mediterranean Sea, and four for the East Mediterranean Sea. The high number of new findings is attributed to the sampling methods applied, the under- or unsearched marine habitats investigated, the different types of substrates and depths covered, and the multilateral co-operation.

## Methods

The collection of specimens was conducted from October 2008 to February 2018 in various locations of Greece, according to Manousis and Galinou-Mitsoudi [59]. For each specimen collected, the following data have been recorded: location, depth, type of habitat/substrate and size (length, unless otherwise stated). For the species nomenclature update (February 2018) besides the Marine Biodiversity and Ecosystem Functioning EU Network of Excellence [60] and World Register of Marine Species [61], the Taxonomic on-line Database on European Marine Mollusca [62] was also used. In addition, the Hellenic Network on Aquatic Invasive Species [63] and the Marine Mediterranean Invasing Alien Species database [64] were used for the alien species status in the Hellenic and Mediterranean Seas. Protoconch whorls were counted according to Verduin [65], the measurement of the protoconch dimensions was performed at side view with its transition to the teleoconch at the far left, the measurement of the protoconch maximum diameter was performed at top view according to Gofas and Oliver [66]. The slenderness (h/w) of the shell was estimated including the outer lip of the aperture in the shell’s width.

The specimens are deposited in the private collections of Dr. Manousis, Mr. Kontadakis, Mr. Mbazios and Mr. Polyzoulis. Scientists are welcome to have access to the biological material for observation after arrangement.

## Abbreviations

CCK: Collection Constantinos Kontadakis
CDM: Collection Dimitris Manios
CTM: Collection Thanasis Manousis
CGMb: Collection George Mbazios
sh.: shell(s)
CGP: Collection George Polyzoulis
CGZ: Collection Giannis Zaminos
CPO: Collection Panagiotis Ovalis
l.i.: live individual
